# Resequencing and Comparative Genomics of *Stagonospora nodorum*: Sectional Gene Absence and Effector Discovery

**DOI:** 10.1534/g3.112.004994

**Published:** 2013-06-01

**Authors:** Robert Andrew Syme, James K. Hane, Timothy L. Friesen, Richard P. Oliver

**Affiliations:** * Australian Centre for Necrotrophic Fungal Pathogens, Curtin University, Department of Environment and Agriculture, Bentley WA 6845, Australia; † Molecular Pathology and Plant Pathology Laboratory, Centre for Environment and Life Sciences, CSIRO, Floreat WA 6014, Australia; ‡ U.S. Department of Agriculture, Agricultural Research Service, Cereal Crops Research Unit, Fargo, North Dakota 58102-2765

**Keywords:** Fungal comparative genomics, Fungal effectors, Phytopathogen

## Abstract

*Stagonospora nodorum* is an important wheat (*Triticum aestivum*) pathogen in many parts of the world, causing major yield losses. It was the first species in the large fungal Dothideomycete class to be genome sequenced. The reference genome sequence (SN15) has been instrumental in the discovery of genes encoding necrotrophic effectors that induce disease symptoms in specific host genotypes. Here we present the genome sequence of two further *S. nodorum* strains (Sn4 and Sn79) that differ in their effector repertoire from the reference. Sn79 is avirulent on wheat and produces no apparent effectors when infiltrated onto many cultivars and mapping population parents. Sn4 is pathogenic on wheat and has virulences not found in SN15. The new strains, sequenced with short-read Illumina chemistry, are compared with SN15 by a combination of mapping and de novo assembly approaches. Each of the genomes contains a large number of strain-specific genes, many of which have no meaningful similarity to any known gene. Large contiguous sections of the reference genome are absent in the two newly sequenced strains. We refer to these differences as “sectional gene absences.” The presence of genes in pathogenic strains and absence in Sn79 is added to computationally predicted properties of known proteins to produce a list of likely effector candidates. Transposon insertion was observed in the mitochondrial genomes of virulent strains where the avirulent strain retained the likely ancestral sequence. The study suggests that short-read enabled comparative genomics is an effective way to both identify new *S. nodorum* effector candidates and to illuminate evolutionary processes in this species.

*Stagonospora nodorum* (teleomorph: *Phaeosphaeria nodorum*; *syn. Septoria*) is the causal agent of the wheat (*Triticum aestivum*) diseases Stagonospora nodorum blotch and glume blotch (Solomon *et al.* 2006a,b; Oliver *et al.* 2012). *S. nodorum* is an economically important pathogen in many parts of the world, causing major yield losses and degradation of grain quality (Murray and Brennan 2009; Tan *et al.* 2009).

The genome sequence of a reference strain (SN15) was shotgun-sequenced using Sanger technology and assembled into 107 nuclear scaffolds and the complete mitochondrial genome (Hane *et al.* 2007). The nuclear scaffolds totaled 38 Mb and included approximately 5% long interspersed repeats. Of the 15,980 genes initially predicted to be present in the SN15 nuclear assembly, 12,382 have had their annotation manually corrected or presence validated (Bringans *et al.* 2009; Casey *et al.* 2010; Ipcho *et al.* 2012).

A major impact stemming from the genome sequence was the identification of necrotrophic effector genes (previously called host-specific or host-selective toxins). These studies revealed that disease symptoms and severity differed in a cultivar- and isolate-specific manner. It therefore has become a priority to sequence more isolates to determine the extent of genetic variation. Necrotrophic effectors have been characterized in *S. nodorum* by a combination of approaches that typically start with the mapping of disease quantitative trait loci (QTL). More than 20 distinct disease resistance/susceptibility QTL have been mapped in wheat and the number continues to grow (Friesen *et al.* 2007; Abeysekara *et al.* 2009; Faris and Friesen 2009; Friesen *et al.* 2009; Chu *et al.* 2010; Abeysekara *et al.* 2012; Crook *et al.* 2012; Liu *et al.* 2012; Oliver *et al.* 2012). The isolation of effector-active proteins either by direct purification from culture filtrates or by expression of candidate effector genes in microbial systems allows the mapping of sensitivity loci in structured wheat populations. Co-location of effector sensitivity loci and disease QTL has been performed in five cases. Three of the effectors have been cloned and fully characterized. These are *SnToxA*, *SnTox1*, and *SnTox3* (Friesen *et al.* 2006; Liu *et al.* 2009; Liu *et al.* 2012). Each of these is a small, secreted, and cysteine-rich protein with important disulfide bonds (Luderer *et al.* 2002; Liu *et al.* 2009). In addition, two of the effector genes (*SnToxA* and *SnTox3*) were found to be adjacent to repetitive DNA or to scaffold termini.

Different strains of *S. nodorum* produce different suites of effectors. Effector gene absence is a commonly observed allele in the three well-known effectors produced by *S. nodorum*. Haplotype analysis of worldwide populations of *S. nodorum* shows 15% of isolates lack *SnTox1* (Liu *et al.* 2012), 40% of isolates lack *SnTox3* (Liu *et al.* 2009), and 60% of isolates lack *SnToxA*, (Stukenbrock and Mcdonald 2007). Some strains produce no apparent effectors. Effector-deficient strains are avirulent on wheat (Friesen *et al.* 2006; Liu *et al.* 2009; Zhang *et al.* 2011; Liu *et al.* 2012) but cause disease on related grass weeds.

The three known effectors exist in a number of variant forms expressed from up to 13 different alleles. Differences in the protein sequence modulate effector activity (Tan *et al.* 2012). The sequence differences carry the hallmark of accelerated evolution as determined by elevated dN/dS ratios (Stukenbrock and Mcdonald 2007).

The related wheat pathogen *Pyrenophora tritici-repentis* also produces a battery of effectors. The majority of isolates produce a version of ToxA, here referred to as *PtrToxA* (Ciuffetti *et al.* 1997; Friesen *et al.* 2006). We presented several lines of evidence that a *ToxA* gene in *S. nodorum* was laterally transferred to *P. tritici-repentis* shortly before 1941 (Friesen *et al.* 2006). The *SnToxA* sequence in *S. nodorum* was present on a scaffold of 32.4 kb. A central section of 11 kb containing *SnToxA* was highly homologous and colinear with the *PtrToxA* containing sequence. The 11 kb also contained a gene for a DNA transposase. Repetitive DNA was found on either side of the 11 kb whereupon an easily recognized level of homology was lost. This finding suggested that the *ToxA* gene was transferred along with at least 11 kb of DNA and possibly as part of a transposon (Friesen *et al.* 2006). The publication of the *P. tritici-repentis* Pt-1c genome (Manning *et al.* 2013) and the sequences of strains of both species that lack *ToxA* allows us to examine this phenomenon in more detail.

In this study we have used second-generation sequencing to determine the DNA sequence of two further strains of *S*. *nodorum*. One strain—Sn4—was isolated from wheat in the United States. It is known to produce a different complement of effectors to SN15. The second strain, Sn79-1087 (hereafter Sn79), was isolated from the grassy weed *Agropyron* (Friesen *et al.* 2006). The Sn79 strain is essentially avirulent on wheat and Sn79 culture filtrates produce no reactions on a diverse panel of wheat cultivars (Figure 1).

**Figure 1:**
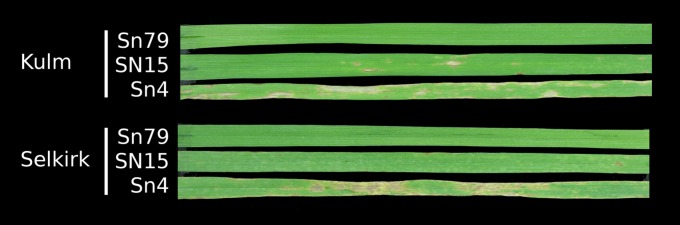
The wheat varieties Kulm and Selkirk infected with *S. nodorum* strains Sn79, SN15, and Sn4.

Comparison of the genome sequences of these two additional *S. nodorum* strains was undertaken to identify further effectors present in Sn4 or SN15 but absent in Sn79 and to explore the genetic differences in each strain that underpins their differing pathogenicity profiles.

## Materials and Methods

### Strains, infection, DNA preparation, and sequencing

*S. nodorum* strains Sn4 (Zhang *et al.* 2011) and Sn79 (Friesen *et al.* 2006) were grown in Fries3 with 30 mM glutamate for 1 wk. Spores were harvested and used to infect detached leaves of wheat cultivars Kulm and Selkirk as previously described (Friesen *et al.* 2006). For DNA extraction, mycelium was harvested with a milk filter and cells lysed with a bead crusher. The concentrations of DNA extracted using the CTAB protocol (Doyle and Doyle 1987) were measured to be 364 ng/mL for the Sn4 extraction and 223 ng/mL for Sn79.

### Mapping

Raw reads were filtered using one round of cutadapt v1.0 (Martin 2011) using a Phred quality threshold of 28, trimming known Illumina adapters from the 3′ end. Trimmed reads were mapped to the reference sequence using BWA 0.5.7 (Li and Durbin 2009), allowing for three gap openings and 21-bp gap extensions per read to allow for large indels. Relatively loose mapping parameters were chosen to reduce the probability of false positive low-coverage regions. Reads mapping around identified indels were locally realigned with GATK 1.2.4 (McKenna *et al.* 2010) using the RealignerTargetCreator and IndelRealigner modules to minimize the likelihood of false single-nucleotide polymorphisms (SNPs).

Mapping depth overviews were visualized as Hilbert curves using the HilbertVis v1.15 (Anders 2009), Rsamtools 1.0, and Bioconductor 2.6 (Gentleman *et al.* 2004) packages. Details of the Hilbert curve generation are available in Supporting Information, Figure S1. Regions of low coverage and consecutive gene absence were calculated using the genomeCoverageBed and intersectBed utilities of BEDTools 2.12.0 (Quinlan and Hall 2010). Regions of low coverage were defined as parts of the reference genome with mapped read depth of 5 or less. Scaffold gaps of poly-‘N’ in the reference sequence were removed from the list of low coverage regions as no reads can map to these loci.

### Assembly

In addition to mapping the reads from Sn4 and Sn79 back to the reference SN15 genome, each genome was assembled *de novo* with Velvet v1.0.10 (Zerbino and Birney 2008) using VelvetOptimizer 2.2.0 to find the assembly with optimal N50 length.

### Mitochondrial sequence

The mitochondrial genomes for Sn4 and Sn79 were considered separately to the nuclear genome. Sequences for the new mitochondrial genomes were inferred from the consensus of reads mapped by BWA to the SN15 mitochondrial genome. Two instances of manual gap removal and contig joining were required to finish the assembly. The velvet assembly was cleaned of mitochondrial sequence by aligning the velvet contigs to the SN15 reference with Mauve 2 using default parameters and removing contigs that aligned with the mitochondrial sequence. The removed mitochondrial contigs were manually checked for major structural changes not present in BWA-mapped consensus to validate the consensus assembly.

### Annotation

Draft gene models were generated with MAKER2 (Cantarel *et al.* 2008) using evidence from a SNAP (version 2010-07-28) hidden Markov model generated from the validated SN15 gene structures, a Genemark ES 2.3 hidden Markov model (Ter-Hovhannisyan *et al.* 2008) trained on each of the new genome sequences, and mRNA sequences from the set of validated SN15 genes. Complete predicted proteins were clustered with the validated SN15 reference proteins using OrthoMCL 2.0.2 (Li *et al.* 2003), using an mcl expansion parameter of 1.5 and a maximum evalue of 1e-5 for the blastp search v2.2.27+.

### Repeats

Known *Stagonospora* repeats (Hane *et al.* 2007; Hane and Oliver 2008; Hane and Oliver 2010); transposons Molly [Genbank:AJ488502.1], Pixie [Genbank:AJ488503.1], and Elsa [Genbank:AJ277966.1]; and simple repeats were identified using RepeatMasker v3.2.8 and crossmatch (from phrap/cross_match/swat ver 1.090518) (Smit *et al.* 1996-2004) in the Sn4 and Sn79 genome sequences using default parameters.

### Repeat-induced point mutation (RIP) analysis

RIP is a fungal-specific process in which duplicated sequences are mutated during meiosis (Selker *et al.* 1987; Selker 1990). In Pezizomycotina, RIP modifies CpN dinucleotides and changes are predominantly in the direction CpA to TpA (Cambareri *et al.* 1989; Clutterbuck 2011). RIPCAL identifies regions likely to have undergone RIP by alignment of similar sequences and scanning for nucleotide changes that are characteristic of RIP (Hane and Oliver 2010). Thus, RIPcal can be used to identify both the presence and direction of a RIP-like sequence change. *S. nodorum* SN15 scaffolds were identified that showed sequence similarity to the *ToxA* region in *P. tritici-repentis* by blat v34 (Kent 2002) using parameters minIdentity = 70 and minScore = 200. For each blat matched region, the nucleotide sequence was extracted from each species’ scaffold and aligned with ClustalW v2.1 (Larkin *et al.* 2007). A 250-bp nonoverlapping sliding window was moved across the concatenated alignments and RIP-like mutations of CpN → TpN were identified from *Pyrenophora* to *Stagonospora* and then from *Stagonospora* to *Pyrenophora* using RIPCAL.

### Phylogenetic analysis

*S. nodorum* Sn4, Sn79, SN15, *P. tritici-repentis*, and *L. maculans* protein sequences for the AFTOL2 (Celio *et al.* 2006) genes *CDC47*, *KRR1*, *CCT4*, *GCD6*, *GPI8*, *MCM3*, *COX15*, *RAD3*, *DBP3*, *NBP35*, *CHC1*, and *RBG2* were identified in the resequenced strains using blast. Homologous genes from the three strains were aligned using MUSCLE 3.6 (Edgar 2004) using default parameters. The alignments were then manually trimmed and concatenated to provide regions present in all five genomes. The concatenated alignments were used to construct phylogenetic trees using the neighbor joining algorithm provided by Geneious (Drummond *et al.* 2011), using the Jukes-Cantor model for genetic distance and *L. maculans* as an outgroup. A consensus tree from 20 replicates was built using a support threshold of 50%.

### Effector candidates

Each of the validated SN15 proteins was assessed against a series of normative assumptions about the characteristics of proteinaceous effectors (Table 1). Small proteins (≤30 kD, calculated using BioRuby 1.4.2 (Goto *et al.* 2010) were scored. Mean cysteine content as a percentage of total protein length was calculated and proteins were scored if they had cysteine percentage greater than one standard deviation from the mean. Proteins were scored if they were encoded by genes within 5 kb of repeats. Repetitive sequences are likely to result in contig breaks in the final assembly, so scaffold ends were treated as equivalent to repeats for this scoring step. Proteins were scored if they had no blast hits to the National Center for Biotechnology Information nonredundant protein database (NR) after we excluded *S. nodorum* proteins, using a minimum e-value cutoff of 1e-20. Proteins were scored if WolfPSort v0.2 (Horton *et al.* 2007) predicted the protein to be located extracellularly, or SignalP 3.0 (Bendtsen *et al.* 2004) identified a signal peptide in the sequence. WolfPSort was run with “fungi” as the organism source. SignalP was run with default parameters. Proteins with coding sequence coverage by Sn4 reads of at least 20% were scored, and proteins with coding sequence coverage by Sn79 reads <20% were scored. Evidence of positive selection for each gene was calculated by taking Sn4 SNPs identified in coding regions and measuring dN/dS using the CNFGTR model (Yap *et al.* 2010) as implemented in PyCogent 1.5.1 (Knight *et al.* 2007).

**Table 1:** Criteria used to identify effector candidates

Criteria	*S. nodorum sn15* Proteins That Match the Criteria
*In silico* analysis	
Protein size < 30 kDa	7069
Cysteine rich*^a^*	1817
Within 5 kb of repeats or scaffold ends	9119
No blasts matches in nr (evalue < 1e-5)	2650
Predicted to be secreted by WolfPSort or SignalP	2788
Resequencing data	
Present, but modified coding sequence in sn4.	6507
Absent in Sn79 (read coverage of coding sequence <20%)	1943
Evidence of positive selection pressure	729

a Proteins with cysteine composition percentage more than one SD above the mean.

## Results

### Cultivar response

We chose two strains for resequencing. Sn79 was isolated from Agropyron and produces no significant disease on any wheat cultivar tested (Figure 1). Infiltration of Sn79 culture filtrate into wheat cultivars produced no reaction but transformation of ToxA, Tox1, or Tox3 into Sn79 rendered the strain capable of causing disease and necrosis on wheat lines carrying sensitivity loci for the respective effector (Friesen *et al.* 2006; Liu *et al.* 2009, 2012). Sn4 is a virulent wheat isolate that is known to produce additional effectors compared with SN15 (Faris *et al.* 2011). Figure 1 shows the more pronounced necrosis in response to Sn4 than both SN15 and Sn79 on wheat cultivars Kulm and Selkirk which suggests the presence of effectors in Sn4 that are absent from SN15.

### Sequencing

DNA sequencing of 35-bp and 75-bp single-end Illumina read libraries yielded 7,097,630 and 13,783,290 reads, respectively, for strain Sn4 and 6,685,623 reads and 15,615,628 reads for Sn79. Assuming the newly sequenced genome sizes are equal to that of SN15 assembly (37.2 Mbp), the sequencing data provided 34-38x coverage.

The reads for each new strain were assembled using Velvet final contigs are available from NCBI as bioproject accessions PRJNA170815 (Sn4) and PRJNA170816 (Sn79). A summary of the genome assembly and mapping statistics is given in Table 2. As expected, the Illumina-sequenced genomes were more fragmented than the Sanger-sequenced reference strain. Commonly reported metrics of assembly contiguity are N50 and L50 (N50 length). The N50 is the smallest number of sequences that contain 50% of the total assembly length and the L50 is the length of the smallest sequence within this subset. The fragmentation of the short-read assemblies has resulted in L50s of 22 kbp and 17 kbp for Sn4 and Sn79 compared with 1.045 Mbp for SN15 and contig counts of 2559 and 3132 for Sn4 and Sn79 compared to 107 scaffolds for SN15. The assembly sizes of Sn4 and Sn79 are comparable (34.6 Mbp and 33.8 Mbp) but smaller than SN15 (37.2 Mbp) because of reduced representation of their repetitive DNA content.

**Table 2:** Genome overview. The resequenced genome assemblies are rich in genic regions

	*S. nodorum* SN15*^a^*		*S. nodorum* Sn4	*S. nodorum* Sn79
Nuclear assembly				
Sequencing technology	Sanger		Illumina	Illumina
Assembled size, Mbp	37.2		34.6	33.8
L50, kbp	1,045		22	17
N50 (scaffolds or contigs)	13		499	613
Max, kbp	2532		113	88
Scaffolds >1kb	107		2559	3132
G+C content, %	50.5		51.7	51.9
	Validated*^b^*	Nonvalidated		
Predicted protein coding gene number	12,382	3598	14,391	14,352
Mean protein size (amino acids)	423	232	437	434
Median protein size (amino acids)	347	170	367	366
Mean exons/gene	2.65	2.30	2.45	2.45
Mean exon length, bp	480	306	515	510
Median exon length, bp	278	170	293	296
Gene density (genes per 10 kb)	3.3	−	4.2	4.2
Mitochondrial genome				
Size, kbp	49.8	−	49.8	42.4
G+C content, %	29.4	−	29.4	26.9
Gene content	22	−	22	19

a Hane *et al.* 2007.

b Genes validated by expressed sequence tagging, proteogenomics, and microarray evidence.

The process of *de novo* assembly with short reads is likely to collapse highly similar repeat units and assemble only divergent repeat copies as well as single copy regions. Fragments of known *S. nodorum* repeats (Hane *et al.* 2007; Hane and Oliver 2008, 2010), were identified in the new assemblies. The short-read assemblies contain 8.5% (Sn4) and 8.4% (Sn79) of the total repeat content of SN15 (data not shown). All but one of the SN15 repeat families was detected as fragments in the new assemblies. The exception was the telomere-associated repeat R22, which was not detected in the Sn4 assembly. The SN15 genome contains 35.48 Mbp of nonrepetitive nuclear DNA, comparable with the assembly sizes of Sn4 (34.6 Mbp) and Sn79 (33.8 Mbp). We therefore tentatively conclude that all three genomes have similar genome sizes.

### Gene calling

We have accumulated substantial evidence for the validation of 12,383 genes in SN15 (Bringans *et al.* 2009; Casey *et al.* 2010; Ipcho *et al.* 2012) and demoted 3598 genes to “nonvalidated” status (Hane *et al.* 2007). Gene calling by GeneMark-ES v2 (Ter-Hovhannisyan *et al.* 2008) in the new assemblies predicted 14,391 and 14,352 genes in Sn4 and Sn79, respectively. The genes in Sn4 and Sn79 have average feature sizes comparable with the validated SN15 genes and much larger than the discarded SN15 genes (Table 2). Compared with experimentally validated gene models in SN15, the gene models in Sn4 and Sn79 have a slightly larger mean protein size (437aa and 434aa compared with 423aa), a larger median protein size (367aa and 366aa compared with 347aa), fewer exons/gene (2.45 compared with 2.65), and longer mean exon length (515 bp and 510 bp compared with 408 bp). These findings indicate that the new assemblies and gene calls are of comparable accuracy to the SN15 reference and are likely better than the set of low-confidence unvalidated reference genes.

**Figure 2:**
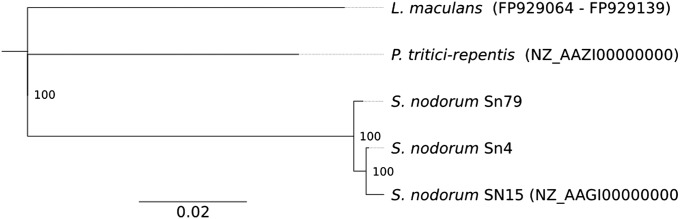
Phylogenetic tree constructed from an alignment of the AFTOL proteins CDC47, KRR1, CCT4, GCD6, GPI8, MCM3, COX15, RAD3, DBP3, NBP35, CHC1, and RGB2. Genbank accessions are shown in brackets and consensus support (%) shown at tree nodes.

The phylogenetic relationships of the three strains were investigated by comparing 15 single-copy or low-copy genes used routinely for phylogenetic analysis. These genes were aligned together with those of *P. tritici-repentis* and *L. maculans*, which constituted an out-group. The phylogenetic tree indicates that each new strain is closely related to SN15 and thus a *bona fide S. nodorum* sequence. It also indicated that the two wheat pathogens are more closely related to each other than either is to the grass pathogen Sn79 (Figure 2). Tree topologies of the individual genes agreed with the tree constructed from the sequence concatenation (data not shown). The three strains are equally distant from both species in the outgroup. This finding is consistent with recent molecular analyses of Pleosporales that place *Pyrenophora*, *Leptosphaeria* and *Phaeosphaeria* (*Stagonospora*) in three distinct families within the suborder Pleosporinae (Zhang *et al.* 2009). Previous authors had placed *S. nodorum* in the *Leptosphaeria* genus.

### Comparative genomics

The presence and absence of regions in the SN15 genome was analyzed in strains Sn4 and Sn79 via a combination of read-mapping and *de novo* assembly. *De novo* assembly was important for the detection of loci that were present in Sn4 or Sn79 but absent in SN15 and thus not detectable by read-mapping.

The new reads were mapped to the genome by BWA. The average mapped read depth in coding regions were 23.2X (Sn4) and 26.2X (Sn79) but was highly variable. Repetitive regions were unreliably accounted for by this procedure, so we only consider low copy number, mainly genic regions here.

Mapping of the reads of each strain to the reference assembly and calculating coverage of each gene’s coding sequence suggested that 180 SN15 genes were absent from Sn4 and 367 SN15 genes were absent from Sn79 (Table 3) where genes with <5% coding region coverage are defined as absent. Inspection of the pattern of missing genes suggested that in many cases contiguous stretches of genes were absent. Quantification of this phenomenon showed that 57% (Sn4) and 76% (Sn79) of absent genes have a neighboring gene that is also absent. We refer to the contiguous runs of missing genes as sectional gene absence (SGA). The degree of SGA was much greater in Sn79 in both frequency and size. The largest SGA from Sn4 included only five genes, and the largest from Sn79 included 51 genes.

**Table 3:** *S*. *nodorum* SN15 genes deemed absent in the newly sequenced strains

	*S. nodorum Sn4*	*S. nodorum Sn79*
SN15 genes absent in the other strains (<5% exon coverage)	180	367
SN15 genes absent from other strain in sections	103 (23 with informative blast)	279 (74 with informative blast)
Largest sectional gene absence (gene count)	10	51
Number of SN15 sections absent from other strain	40	56

Illumina reads from Sn4 and Sn79-1087 were mapped to the SN15 scaffolds and genes with <5% coding sequence coverage were identified as absent. Few genes are absent in isolation, and many are missing from Sn79 in large sections.

Genes sectionally absent from Sn79 include all the genes on scaffold 44 (48 genes) and scaffold 45 (51 genes, the largest SGA), and the polyketide synthase gene SNOG_07866 [NCBI Gene: 5975086], which is absent in a cluster of 11 genes at the end of scaffold 11.

**Figure 3:**
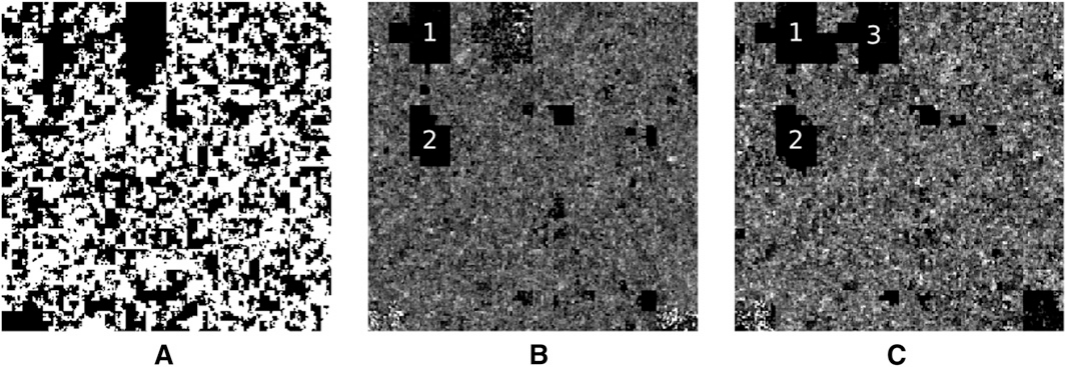
Hilbert plots of coding sequence and read density of SN15 scaffold 2. (A) White regions correspond to coding regions. *S. nodorum* Sn4 (B) and Sn79 (C) mapped read density on SN15 scaffold 2. Lighter shades correspond to higher read density. Regions 1 (45−46 kbp) and 2 (31 kbp) are absent from both Sn4 and Sn79. Region 3 shows a 48-kbp region absent from Sn79.

This pattern of nonrandom SGA prompted us to explore ways to graphically display this finding. Very long read-depth data can be conveniently visualized as a space filling curve that attempts to preserve proximity during its transformation to an increased number of dimensions. Figure 3 shows Hilbert plots generated using R with the HilbertVis (Anders 2009) and Bioconductor (Gentleman *et al.* 2004) packages (see Figure S1 for more information on Hilbert plots). Only scaffold 2 is shown; scaffolds 1–12 are shown in the supplementary data. White sections in Figure 3A represent those sections of SN15 scaffold 2 that are annotated as coding sequence, whereas B and C represent the mapped read density of Sn4 and Sn79, respectively. White regions in B and C are parts of SN15 scaffold 2 that are covered by Sn4 and Sn79. Large dark boxes 1−3 indicate regions of SN15 scaffold 2 that have no reads mapped from Sn4 and Sn79 and are likely to be absent from the newly sequenced strains.

**Figure 4:**
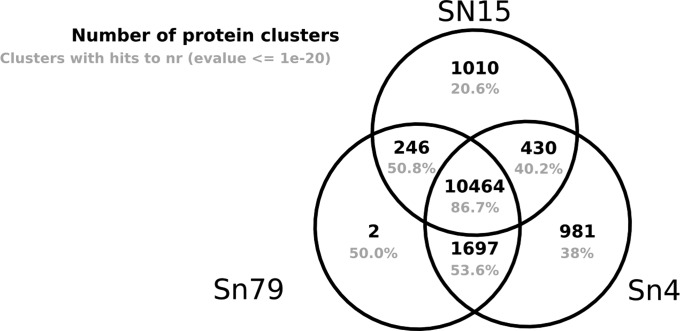
Protein ortholog clusters between the three *S. nodorum* strains. Proteins were grouped into clusters using orthoMCL (Li *et al.* 2003). Clusters were regarded as “nr-unique” if none of its members had blast hits to the nr database at an e-value cutoff of 1e-20.

Assembly of the reads *de novo* allowed us to identify genes not present in the SN15 reference assembly and to estimate the core *S. nodorum* gene set. Clustering the predicted proteins from the three genomes using OrthoMCL gave a core cluster number estimate of 10,464 (Figure 4). The clusters showed a larger conserved gene set between SN15 and Sn4 (430) than between SN15 and Sn79 (246) consistent with the phylogenetic analysis. The large set shared between Sn79 and Sn4 is likely an overestimate as a result of these strains’ shared gene calling procedure. As gene models are improved, the number of genes in this set is likely to fall.

The 10,464 protein clusters in the conserved *S. nodorum* core include many of the products essential to metabolism. Of the protein clusters present in all three strains, 87% had blast hits in NR. A markedly lower percentage of the strain-specific genes (21–50%) had similarity to protein sequences in NR.

Expansion in gene copy number was detected in eight clusters (Table 4). Of the clusters, five contained genes with higher copy numbers in SN15, and three clusters contained genes with greater copy numbers in Sn4. No clusters contained genes expanded in the non-pathogenic strain Sn79. Four of the eight expanded clusters contained genes with no blast hits (e value ≤ 1e–20).

**Table 4:** Gene family expansion in the three *S. nodorum* strains

Cluster id	No. SN15 Proteins	No. Sn4 Proteins	No. Sn79 Proteins	Function
stago_10024	8	0	0	No blast hits with evalue ≤1e-20
stago_10031	1	4	2	Phosphoribosylformylglycinamidine synthase (EC:6.3.5.3)
stago_10129	1	3	1	Conserved with unknown function
stago_10171	4	0	0	No blast hits with evalue ≤1e-20
stago_10172	3	0	0	No blast hits with evalue ≤1e-20
stago_10388	3	0	0	No blast hits with evalue ≤1e-20
stago_10389	3	0	0	Conserved with unknown function
stago_10390	0	3	0	Putative retrotransposon protein

There was no evidence of gene expansion in the non-pathogenic strain Sn79. NB cluster id numbers do not correspond to gene identifiers.

### Effector comparison

**Figure 5:**
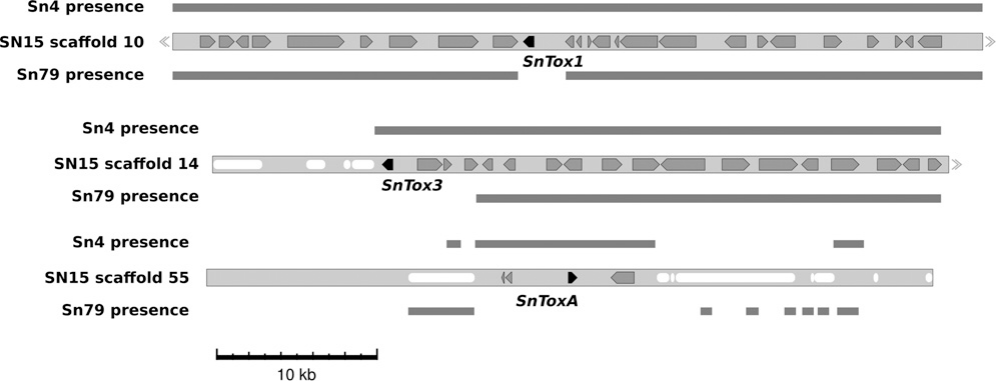
Effector context in three *Stagonospora* strains. Black arrows represent effector genes, gray arrows represent other genes, and white lozenges represent repetitive sequence. Gray boxes above and below the SN15 scaffold sections represent sequence present in the assemblies of the resequenced strains. The sequence surrounding *SnTox1* is present in all three strains. *SnTox3* is absent from Sn79 in a section that includes four genes. The entire *SnToxA* region is absent in Sn79.

*S. nodorum* Sn79 produces none of the three known *S. nodorum* effectors: *SnTox1*, *SnToxA*, and *SnTox3* (Friesen *et al.* 2006; Liu *et al.* 2009, 2012). Effector-containing regions in SN15 were compared with the corresponding regions in Sn4 and Sn79. These known *S. nodorum* effectors are all absent from the Sn79 assembly (Figure 5 and Figure S2). In the case of *SnTox1*, the effector gene and 2 kb of upstream intergenic sequence was absent from Sn79. *SnTox3* was absent from Sn79 together with the three upstream genes (SNOG_08982 to SNOG_08985; NCBI 5976184 to 5976187). The absent genes included a predicted protein-disulfide isomerase, a short-chain dehydrogenase, and a gene without blast hits. The repetitive regions downstream of *SnTox3* gene did not assemble in either Sn4 or Sn79.

**Figure 6:**
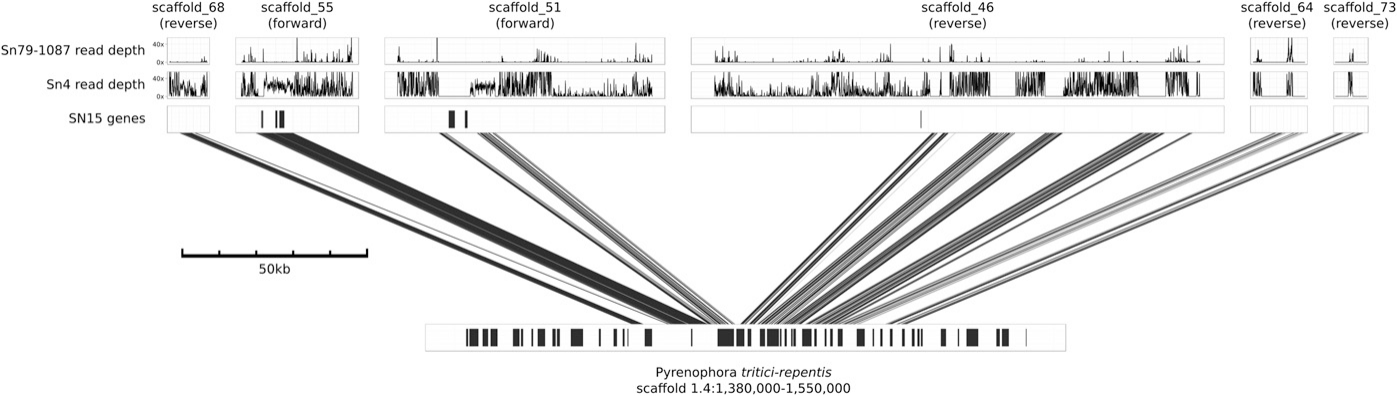
Regions around *ToxA* in *P. tritici-repentis* show homology to other SN15 scaffolds. The scaffolds are linked here with blat hits >75% identity. The total size of the expanded predicted laterally transferred DNA region is at least 72 kb.

*SnToxA* is found on scaffold 55 together with just three more called genes, including a transposase. The remainder of the scaffold comprises sections of gene-free single copy DNA and repetitive elements. The single-copy regions, including a contiguous stretch of 11 kb, were present in Sn4 albeit on different contigs. The 11-kb region was absent in Sn79, but some of the repetitive elements were present on short contigs (Figure 5). The presence of *ToxA* in some strains of both *S. nodorum* and *P. tritici-repentis* is suggestive of lateral gene transfer. We earlier reported that the colinear region of 11 kb found in both SN15 and Sn4 is present in *P. tritici-repentis* with very high levels of similarity (Friesen *et al.* 2006). The assembly of *P. tritici-repentis* Pt-1c using Sanger sequencing aided by optical mapping has recently been published (Manning *et al.* 2013). *PtrToxA* is present on scaffold 4/chromosome 6 of the Pt-1c assembly. Alignment of scaffold 4 to *C. heterostrophus* indicated that a region of 156 kb including *PtrToxA* was absent in the corn pathogen but the homologous flanking regions were present in both species (Manning *et al.* 2013). A series of SN15 scaffolds [68, 55 (including *SnToxA*), 51, 46, 64, and 73] appear to be colinear compared with *P. tritici-repentis* (Figure 6). Regions of colinearity were interspersed with repeated sequences inserted into the SN15 genome. Scaffold 46 has 4 such colinear regions interspersed with five repetitive sections. The total length of the colinear DNA in *P. tritici-repentis* was 72 kb, corresponding to a 350-kb region in SN15 that was inflated by multiple transposon invasions. Read mapping of Sn4 to the SN15 genome indicated that most of the repetitive regions, the 11 kb *SnToxA* region, and a gene free single copy region on scaffolds 68 and 51 were present in Sn4. In contrast, a single copy region on scaffold 51 with two annotated genes, and the single copy regions on scaffolds 46, 64, and 73 were absent in Sn4. In Sn79 all single copy regions were absent and only traces of the repetitive DNA could be found.

**Figure 7:**
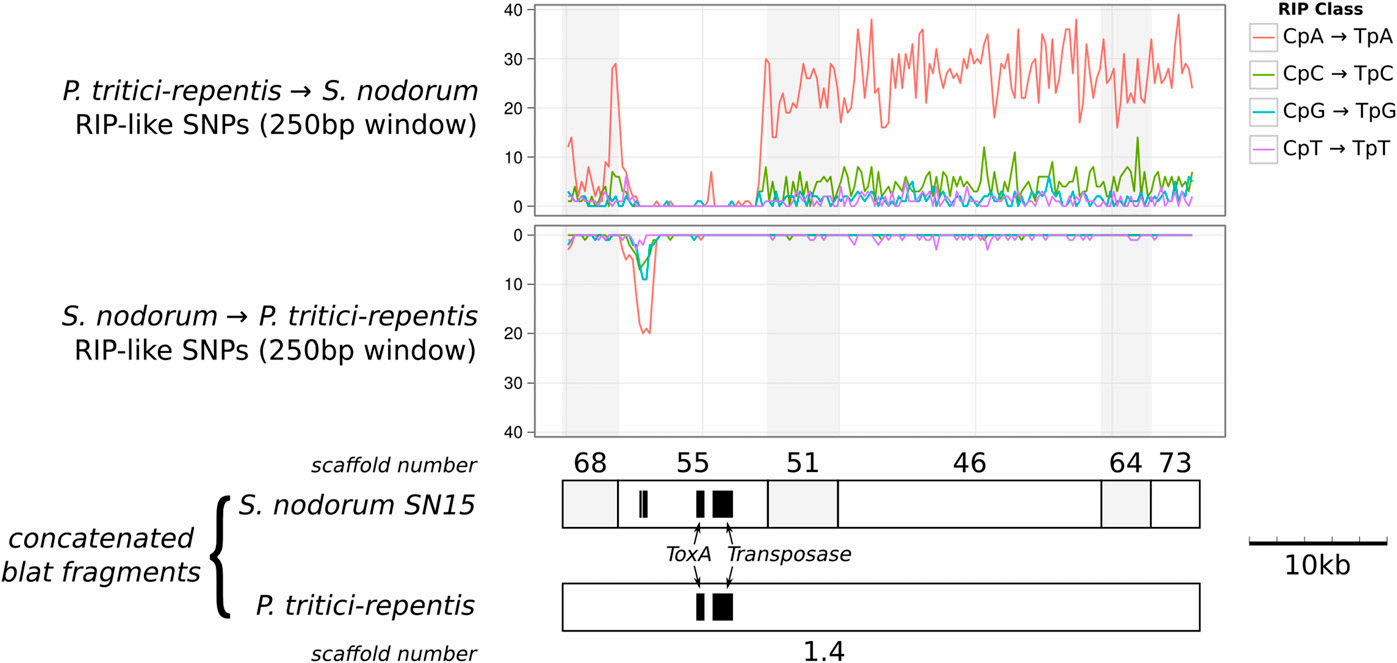
Regions of similarity around ToxA in *P. tritici-repentis* and *S. nodorum* SN15 identified by blat (Figure 6) were concatenated and aligned. RIP irreversibly converts cytosine bases to thymine, therefore comparisons between two sequences have directionality. The frequency of CpN to TpN mutations across 250-bp nonoverlapping windows has been separated by direction into those with CpN dinucleotides of *S. nodorum* being converted into TpN in *P. tritici-repentis* (top) and vice-versa (bottom). CpA to TpA changes that are associated with RIP are indicated by the red line. The location of genes is indicated by black boxes.

The 72 kb of colinear DNA contains 21 genes in *P. tritici-repentis* but only seven genes in SN15. The hallmarks of RIP have been abundantly found in the SN15 genome but only sparsely in the *P. tritici-repentis* genome (Hane and Oliver 2008, 2010; Manning *et al.* 2013). We extracted and aligned 46 kb of matching regions from the 72-kb *ToxA* region shown in Figure 6 and scanned for dinucleotide SNP mutations characteristic of RIP (Figure 7). The dinucleotide changes are predominantly CpA->TpA mutation, which is characteristic of RIP in Pezizomycotina species. Across most of the region, changes are consistent with the *S. nodorum* sequence having been subject to RIP. These changes were most likely in the form of RIP leakage. We have previously observed that RIP-like changes, initiated from interspersed repeat regions, are also found in neighboring single-copy regions (Hane and Oliver 2010; Van de Wouw *et al.* 2010). In contrast, one region upstream of the ToxA gene on scaffold 55 had changes characteristic of RIP in the Pt-1c sequence. Subsequences of this region were found in multiple copies in Pt-1c genome, which suggests that the transferred region has been susceptible to RIP in PTR since its lateral transfer, but the majority has remained in single copy and thus has evaded RIP mutation.

### Mating type locus

**Figure 8:**
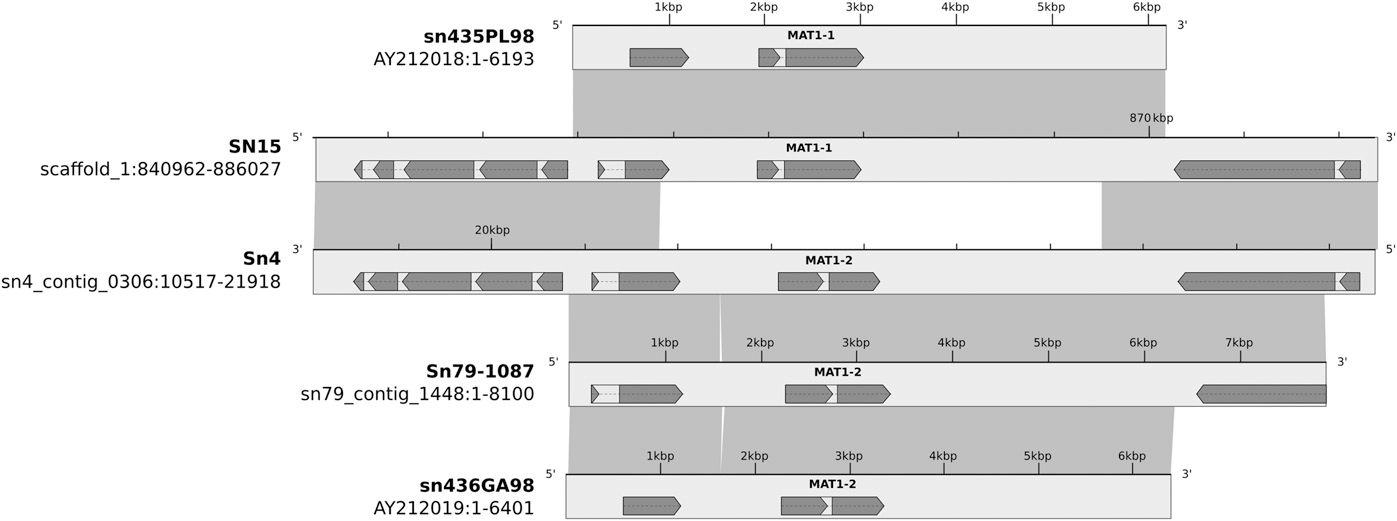
The MAT locus in 5 *Stagonospora* strains showing the two idiomorphs. Strains sn435PL98 and SN15 are mating type 1 (*MAT1-1*) and strains Sn4, Sn79, and sn436GA98 are mating type 2 (*MAT1-2*). Light gray boxes represent nucleotide sequence, and dark gray boxes connect regions with sequence similarity.

The mating type idiotype of SN15 is *Mat1-1* and of Sn4 and Sn79 is *Mat1-2*. These idiomorphs have previously been sequenced (Bennett *et al.* 2003). Sn4 and Sn79 both contained copies of the *MAT1-2* idiomorph (Figure 8). The mating type genes show perfect sequence identity to published sequences. There is a 19-bp region present in the Sn79 *Mat1-2* locus that is absent in both the Sn4 and sn436GA98 sequences.

### Effector candidate selection

The resequencing data were combined with data from existing SN15 gene models to predict likely effector proteins, based on a set of normative assumptions about the qualities of effector molecules and their genes both in *S. nodorum* (Friesen *et al.* 2006; Liu *et al.* 2009, 2012) and other fungal pathogens (De Wit *et al.* 2009) (Table 1). Some criteria were assessed before this sequencing project. These include size, cysteine content, proximity to repetitive elements, and prediction of secretion. Addition of data from this resequencing project allowed us to include criteria reporting gene absence or difference between the three strains.

Because Sn79 lacks detectable wheat effectors, we sought genes that were absent in this strain or highly divergent compared to SN15. Genes with <20% of their coding sequence covered by Sn79 reads (1943 genes) were scored in this criterion. The three known effector genes exist in different isoforms in different strains (Friesen *et al.* 2006; Liu *et al.* 2009, 2012). To exploit this, we sought genes that were present in Sn4 but contained sequence variation compared withSN15; a total of 6507 genes fit this criterion. Sequence comparison of many effector genes shows evidence of accelerated evolution. We therefore screened for genes with elevated dN/dS ratios when comparing Sn4 and SN15. A total of 729 genes showed evidence of high dN/dS.

Each of the criteria matched between 729 and 9119 genes, providing a filter that reduced 12,382 gene models to 159 candidates that matched at least six of the criteria (Table 5). Known effectors *SnTox1* and Sn*ToxA* were ranked first and equal third according to these criteria (Table S1).

**Table 5:** Counts of proteins matching the eight criteria from Table 3

Minimum Effector Criteria Matched	No. sn15 Proteins
8	1
7	10
6	159
5	904
4	3158

The criteria are highly selective for the top effector candidates.

Proteins from the Sn4 genome not observed in SN15 were also scored for likely effector properties including small size (≤30 kD), predicted to be secreted, and cysteine-rich. Of the predicted proteins present in the 981 clusters unique to Sn4, four fulfill all three of the criteria given previously (File S2).

### Mitochondrial genomes

The mitochondrial genomes of Sn4 and Sn79 were considered separately to the nuclear genome. Sequences for the new mitochondria were assembled by taking the consensus sequence of the BWA-mapped reads of the new strains and removing mitochondrial sequences from the Velvet-assembled contigs.

Alignment of the mitochondrial genomes of Sn4 and SN15 showed entirely conserved gene content and order with no indels larger than 3 bp. Two sections of the SN15 and Sn4 mitochondrial genomes were absent in the Sn79 genome (Figure S3). These missing sections included an intronic endonuclease and two downstream open reading frames of unknown function. The total mitochondrial sequence discrepancy was 7.43 kb absent from Sn79 but present in Sn4 and SN15.

## Discussion

Fungal plant pathogenic species are notoriously variable in their morphology and growth characteristics; pathology studies have traditionally focused on differences in virulence and for some species these have been formalized via the definition of *forma specialis*, pathotypes or races. Exploration of the genetics of the virulence differences has proved a fruitful way to understand the molecular bases of virulence properties. Genome sequences of fungal plant pathogens have been obtained for a handful of species using Sanger-based technologies (Oliver *et al.* 2012). The advent of second-generation sequencing technologies has opened up the possibility of sequencing different strains and thereby directly measuring genetic differences.

Early studies of *S. nodorum* readily identified significant genotypic differences in strains based on expressed sequence tag, restriction fragment length polymorphism, and chromosome-length polymorphism studies (Oliver *et al.* 2012) but these differences were not related to virulence properties. It was not until the discovery of necrotrophic effectors that a rational basis for differentiating strains was elaborated (Friesen *et al.* 2008; Tan *et al.* 2010; Oliver *et al.* 2012). Proteins purified from culture filtrates of different isolates induced necrosis in different wheat cultivars. Sensitivity to the proteins mapped to QTL for disease susceptibility that differed between isolates. One strain, isolated from a wild grass, produced neither observable effectors nor disease on wheat. These strains and the new necrotrophic effector rationale provided sufficient incentive for resequencing.

The process of generating second generation sequencing data for two further strains was affordable but presented significant challenges to analyze the data. It was clear that the new strains genomes differed markedly from the reference SN15 strain. These differences were explored by mapping reads onto the reference and by examining *de novo* assemblies.

Both methods showed that many genes differed between the strains or were entirely absent. The mapping data suggests that Sn4/Sn79 lacked at least 180 of 367 genes, respectively, compared with SN15 (Table 3). In addition 6507 Sn4 genes differed in nucleotide coding sequence (Table 1). By any criteria, these are very large strain to strain differences which stretch the definition and technology of resequencing.

The gene complement differences were not randomly located in the genome but were predominantly in the form a contiguous sections containing two to 50 or more absent genes. We refer to this pattern as SGA. These genes include a high proportion without clear homologs in other species. One region absent in Sn79 on scaffold_11:124,000-127,500 comprises a cluster of genes that could well function in synthesizing a secondary metabolite involved in pathogenicity on wheat.

This pattern may resemble the presence in *M. graminicola* and *Fusarium oxysporum* of entire chromosomes that are unevenly harbored by different strains (Ma *et al.* 2010; Goodwin *et al.* 2011). These species contain dispensable chromosomes with relatively low gene density, a high frequency of genes without significant matches to known genes and high repetitive element content. In *S. nodorum* strain-to-strain SGA sometime comprised entire scaffolds. Pulsed field gel and genetic mapping evidence suggests that there are about 20 chromosomes in *S. nodorum* (Cooley and Caten 1991; Malkus *et al.* 2009), whereas the SN15 assembly contains 107 scaffolds. The lack of a finished SN15 genome assembly means that it is not currently possible to determine the chromosomal location of the SGA regions.

Clustering the predicted protein datasets of each strain can estimate the core proteins conserved between them. This approach gave a minimum conserved gene set of 10,464 protein clusters present in all three strains and an additional 430 conserved between both wheat pathogenic strains.

### Mitochondrial sequence

The mitochondrial genomes of Ascomycete fungi contain a well-conserved set of genes but a variable amount of additional DNA (Hane *et al.* 2011). This additional DNA can add 120 kb to the core ca. 40 kb of the genome (Rouxel *et al.* 2011). This additional DNA includes genes or unknown function and intronic genes encoding homing endonucleases. The mitochondrial genome of SN15 is 49,761 bp and includes three open reading frames (ORFs) of unknown function (ORF 1, 2, and 3) and four intronic endonucleases. The mitochondrial genome of Sn4 was found to be essentially identical, but the Sn79 genome differed significantly in one region (Figure S3) lacking the intron found in *atp6* and a region containing ORF 1 and 2. This pattern is consistent with the insertion of ORF3 and three introns into the ancestor of all three strains and the subsequent insertions of the *atp6* intron and the region containing ORF 1 and 2 into the lineage containing Sn4 and SN15. This scenario is consistent with the phylogeny based on nuclear DNA sequences (Figure 2). The detection of the *atp6* intron and the region containing ORF 1 and 2 provides a polar and datable marker for evolutionary studies of the fungus.

### Effector prediction

The main reason for carrying out this study was to facilitate the identification of effectors from the fungal genomes. Experience from a range of pathogens has generated a set of criteria that can be used to select effector candidates for experimental validation. These criteria (Tables 1 and 5) were used to filter and rank 12382 genes so that they can be efficiently prioritized for functional characterization. This process predicted the subsequently verified effector *SnTox1* that conforms to all eight criteria (Liu *et al.* 2012). *SnTox3* and *SnToxA* match 5 and 6 of the criteria respectively indicating that we still have an imperfect knowledge of effectors’ properties.

### Context of effector genes

All three known effector genes were absent from Sn79. Comparisons of the three genomes can be used to infer hypotheses about the evolutionary history of these genes (Figure 5) albeit the short contig lengths limit the conclusions. *SnTox1* is absent from Sn79 but the flanking sequences are homologous in the other two strains. *SnTox3* is absent from Sn79 along with a section of at least three genes. Both of these scenarios are consistent with a horizontal gene transfer hypothesis (Oliver *et al.* 2008) although gene loss from Sn79 is also a possibility; the source of the genes is unknown.

Multiple studies provide evidence supporting recent horizontal transfer for *ToxA* from *S. nodorum* to *P. tritici-repentis* (Friesen *et al.* 2006) and earlier from an unknown source into *S. nodorum* (Stukenbrock and McDonald 2007). The new data in this paper and the *P. tritici-repentis* genome sequence (Manning *et al.* 2013) allow us to speculate on the history of this critical region. Sn79 lacks *ToxA* along with a long section (at least 72 kb) of single copy DNA. This finding suggests that *ToxA* was transferred into the ancestor of both wheat pathogenic strains along with at least this length of DNA (Figure 6). Subsequently, in some strains such as SN15, this section has been invaded by transposons and subjected to RIP (Figure 7). The most parsimonious hypothesis for the source and structure of the *ToxA* region in *P. tritici-repentis* is an *S. nodorum* strain that had not yet suffered this transposon invasion and RIP. The recent acquisition, the homothallic nature of *P. tritici-repentis* and the scarcity of RIP have resulted in the monomorphism of *PtrToxA* (Tan *et al.* 2012) and the retention of genes lost to RIP in *S. nodorum*. The presence of both *ToxA*-expressing and nonexpressing strains of both species in different proportions in different parts of the world (Stukenbrock and McDonald 2007) can be accounted for by asexual propagation, sexual crossing and differential selection by wheat cultivars depending on whether they express the recognition gene *Tsn1* (Oliver *et al.* 2009; Antoni *et al.* 2010; Faris *et al.* 2010). Confirmation and elaboration of these hypotheses must await the assembly of a larger sample of genome sequences from both species.

## Supplementary Material

Supporting Information
