# Supplementary material for: Resequencing and Comparative Genomics of Stagonospora nodorum: Sectional Gene Absence and Effector Discovery
Source: G3 (Bethesda). 2013 Jun 1;3(6):959–69. doi: 10.1534/g3.112.004994 (PMC3689807; doi:10.1534/g3.112.004994)
Supplement: Supporting Information [file supp_3_6_959__index.html]

Resequencing and Comparative Genomics of Stagonospora nodorum: Sectional Gene Absence and Effector Discovery — Supporting Information 

# Resequencing and Comparative Genomics of *Stagonospora nodorum*: Sectional Gene Absence and Effector Discovery

## Supporting Information for Syme *et al.*, 2013

**Files in this Data Supplement:**

- Supporting Information - Figures S1-S3, Files S1-S2, and Table S1 (PDF, 581 KB)
- Figure S1 - An example Hilbert curve showing a gradual change in hue (PDF, 333 KB)
- Figure S2 - Effector context in three *Stagonospora* strains (PDF, 240 KB)
- Figure S3 - A section of the *S. nodorum* mitochondrial genomes showing that a 7667 bp region present in strains SN15 and Sn4 is absent in Sn79 (PDF, 84 KB)
- File S1 - Hilbert Plots (.zip, 19 MB)
- File S2 - sn4 candidates GenBank file (.zip, 2 KB)
- Table S1 - Genes (.xls, 2 MB)
