# Supplementary material for: Resequencing and Comparative Genomics of Stagonospora nodorum: Sectional Gene Absence and Effector Discovery
Source: G3 (Bethesda). 2013 Jun 1;3(6):959–69. doi: 10.1534/g3.112.004994 (PMC3689807; doi:10.1534/g3.112.004994)
Supplement: Supporting Information [file supp_g3.112.004994_004994SI.pdf]

G3 Genes | Genomes | Genetics

Resequencing and comparative genomics of *Stagonospora nodorum*; sectional gene absence and effector discovery

Syme, R. A.[1], Hane, J.K.[2], Friesen, T. L.[3], Oliver, R. P.[1]

[1]Australian Centre for Necrotrophic Fungal Pathogens, Curtin University, Department of Environment and Agriculture, Bentley WA 6845, Australia
[2] Molecular Pathology and Plant Pathology Laboratory, Centre for Environment and Life Sciences, CSIRO, Floreat, Perth, Australia
[3] U.S. Department of Agriculture, Agricultural Research Service, Cereal Crops Research Unit, Fargo, North Dakota 58102-2765, USA

DOI: 10.1534/g3.112.004994

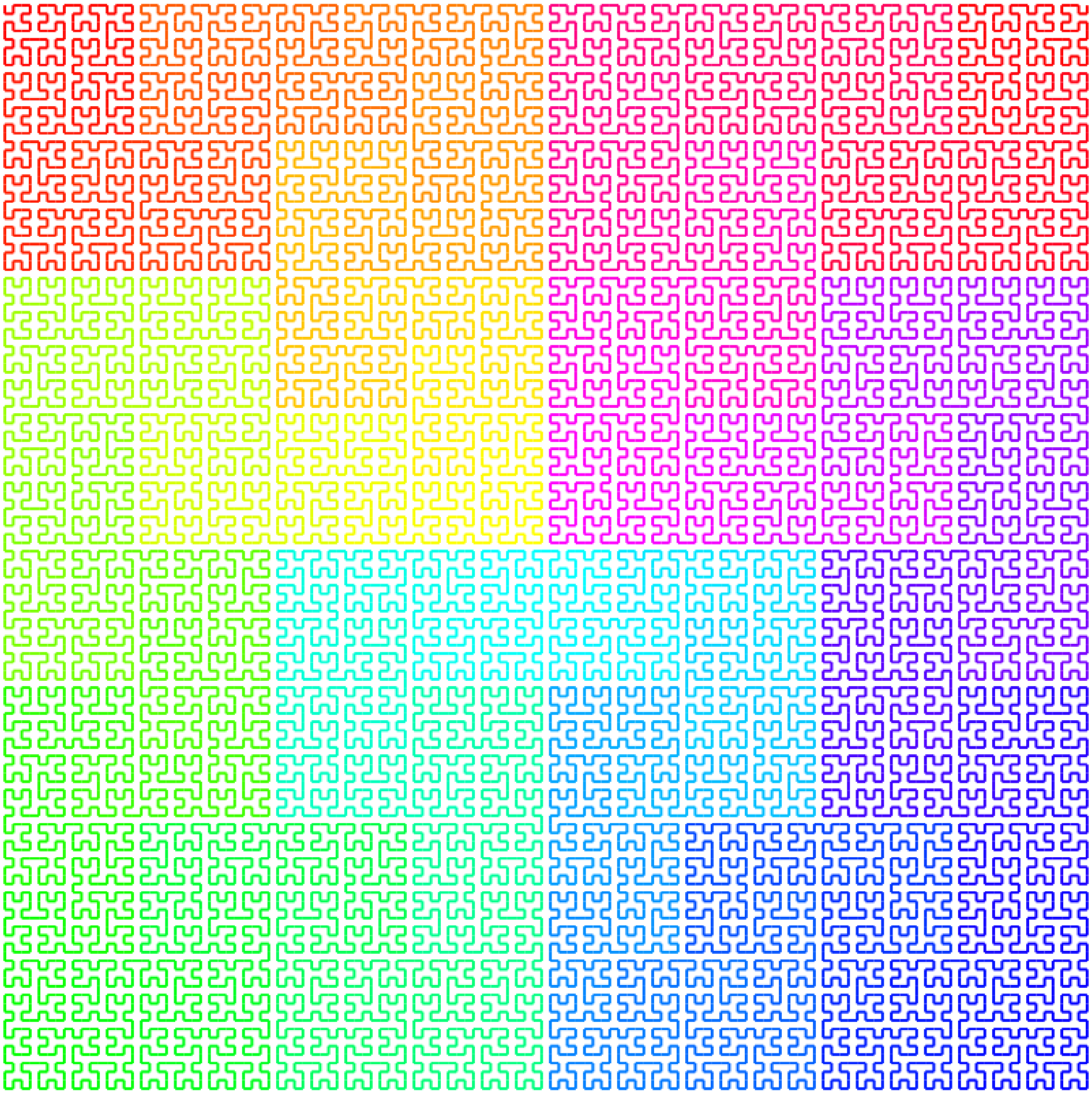

Figure S1 An example Hilbert curve showing a gradual change in hue. The Hilbert curve is an attempt to preserve locality between the original 1 dimensional space and a higher dimensional curve. Any given region in the final plot is likely to be composed of values close together in the original data. In this example, any given region is likely to be composed of a similar colour.

This style of visualisation is helpful when the original data are too large to display in their linear from. Packing the data into a space filling curve allows for a more space-efficient representation of the same information while preserving locality. In a genomic context, it gives the ability to visualise an entire scaffold or chromosome in fairly high resolution. A numeric variable such as read depth can be represented as variations in the lightness of the curve. In this way contiguous features are still visible in the final curve as blocks of a similar shade.

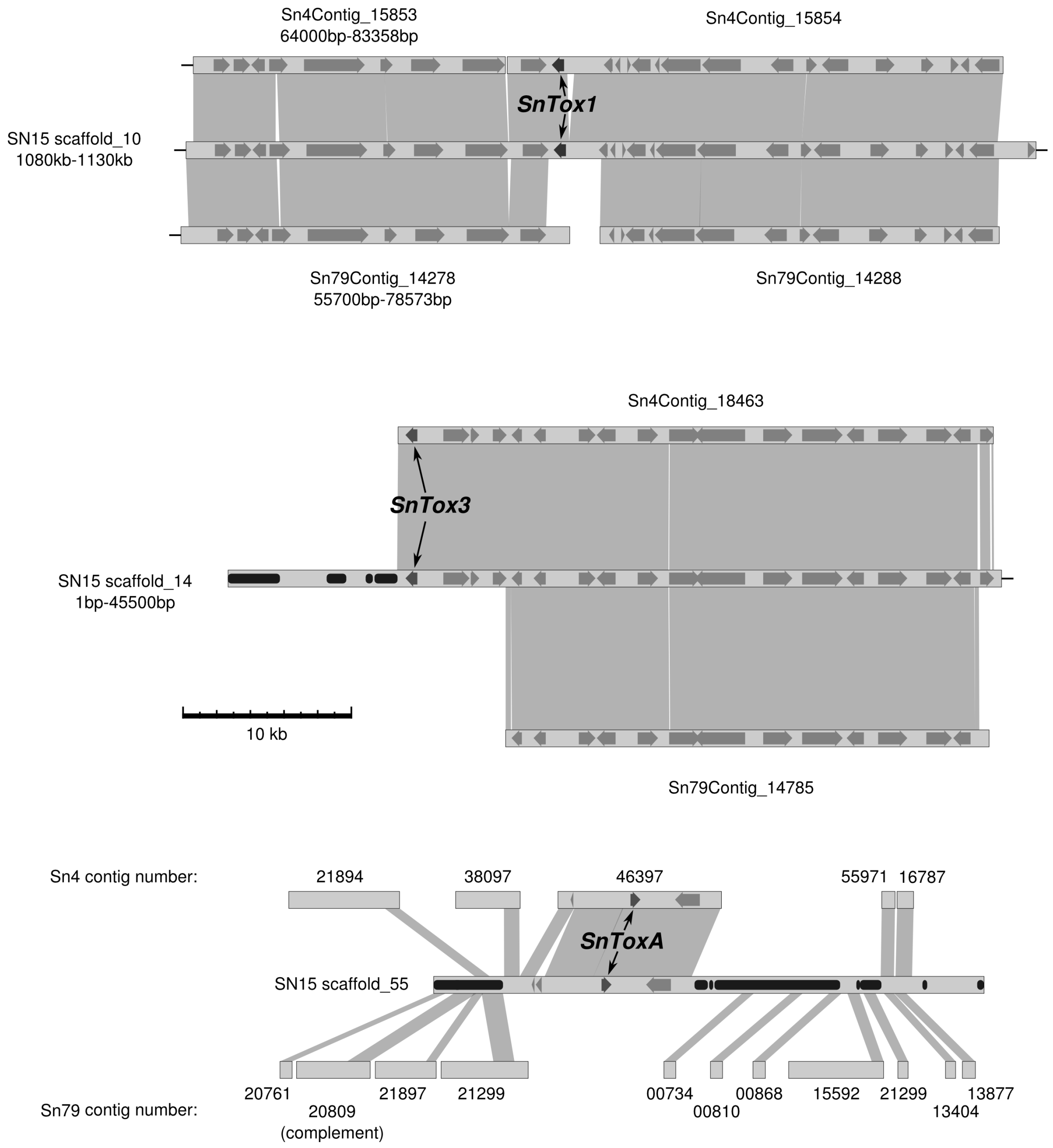

Figure S2 Effector context in three *Stagonospora* strains. Arrows represent genes, lozenges represent repetitive sequence, and boxes represent contigs/scaffolds. Darker trapezoids connecting contigs are blast matches with evalue <= 1e-20. The sequence surrounding *SnTox1* is present in all three strains. *SnTox3* is absent from Sn79is a section that includes four genes. The entire *SnToxA* region is absent in Sn79. Contigs are short around the *SnToxA* region due to the repetitive content in the surrounding sequence.

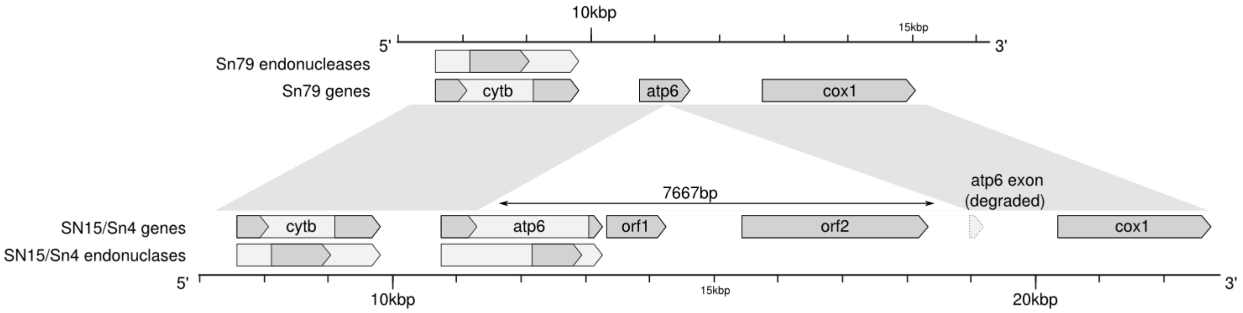

Figure S3 A section of the *S. nodorum* mitochondrial genomes showing that a 7667 bp region present in strains SN15 and Sn4 is absent in Sn79. Regions of homology are shown as darker regions connecting the two sequences. The 7667 bp region includes ORF1 and ORF2, an intronic endonuclease within atp6 and a copy of the 3' *atp6* exon. The insert ends at a degraded copy of the 3' *atp6* exon. This copy differs from the Sn79 *atp6* coding sequence at three SNPs including one nonsense mutation in the degraded Sn15/SN4 copy.

File S1
Hilbert Plots

File S2
sn4 candidates GenBank file

Files S1 and S2 are available for download at http://www.g3journal.org/lookup/suppl/doi:10.1534/g3.112.004994.

Table S1 Genes

Table S1 is available for download at http://www.g3journal.org/lookup/suppl/doi:10.1534/g3.112.004994/-/DC1.
