# Supplementary material for: Resequencing and Comparative Genomics of Stagonospora nodorum: Sectional Gene Absence and Effector Discovery
Source: G3 (Bethesda). 2013 Jun 1;3(6):959–69. doi: 10.1534/g3.112.004994 (PMC3689807; doi:10.1534/g3.112.004994)
Supplement: Supporting Information [file supp_g3.112.004994_FigureS1.pdf]

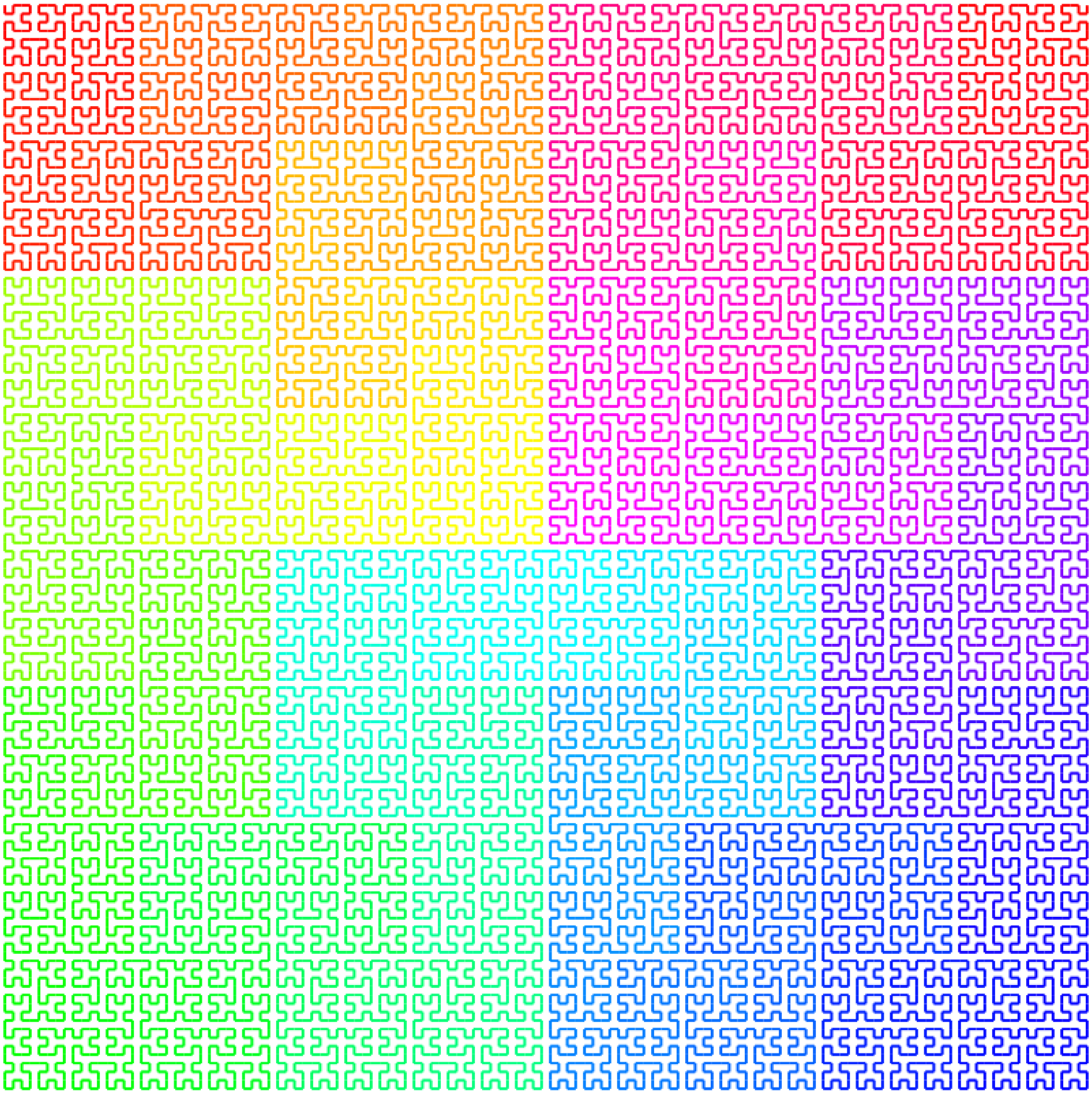

Figure S1 An example Hilbert curve showing a gradual change in hue. The Hilbert curve is an attempt to preserve locality between the original 1 dimensional space and a higher dimensional curve. Any given region in the final plot is likely to be composed of values close together in the original data. In this example, any given region is likely to be composed of a similar colour.

This style of visualisation is helpful when the original data are too large to display in their linear from. Packing the data into a space filling curve allows for a more space-efficient representation of the same information while preserving locality. In a genomic context, it gives the ability to visualise an entire scaffold or chromosome in fairly high resolution. A numeric variable such as read depth can be represented as variations in the lightness of the curve. In this way contiguous features are still visible in the final curve as blocks of a similar shade.
