# Supplementary material for: Resequencing and Comparative Genomics of Stagonospora nodorum: Sectional Gene Absence and Effector Discovery
Source: G3 (Bethesda). 2013 Jun 1;3(6):959–69. doi: 10.1534/g3.112.004994 (PMC3689807; doi:10.1534/g3.112.004994)
Supplement: Supporting Information [file supp_g3.112.004994_FigureS2.pdf]

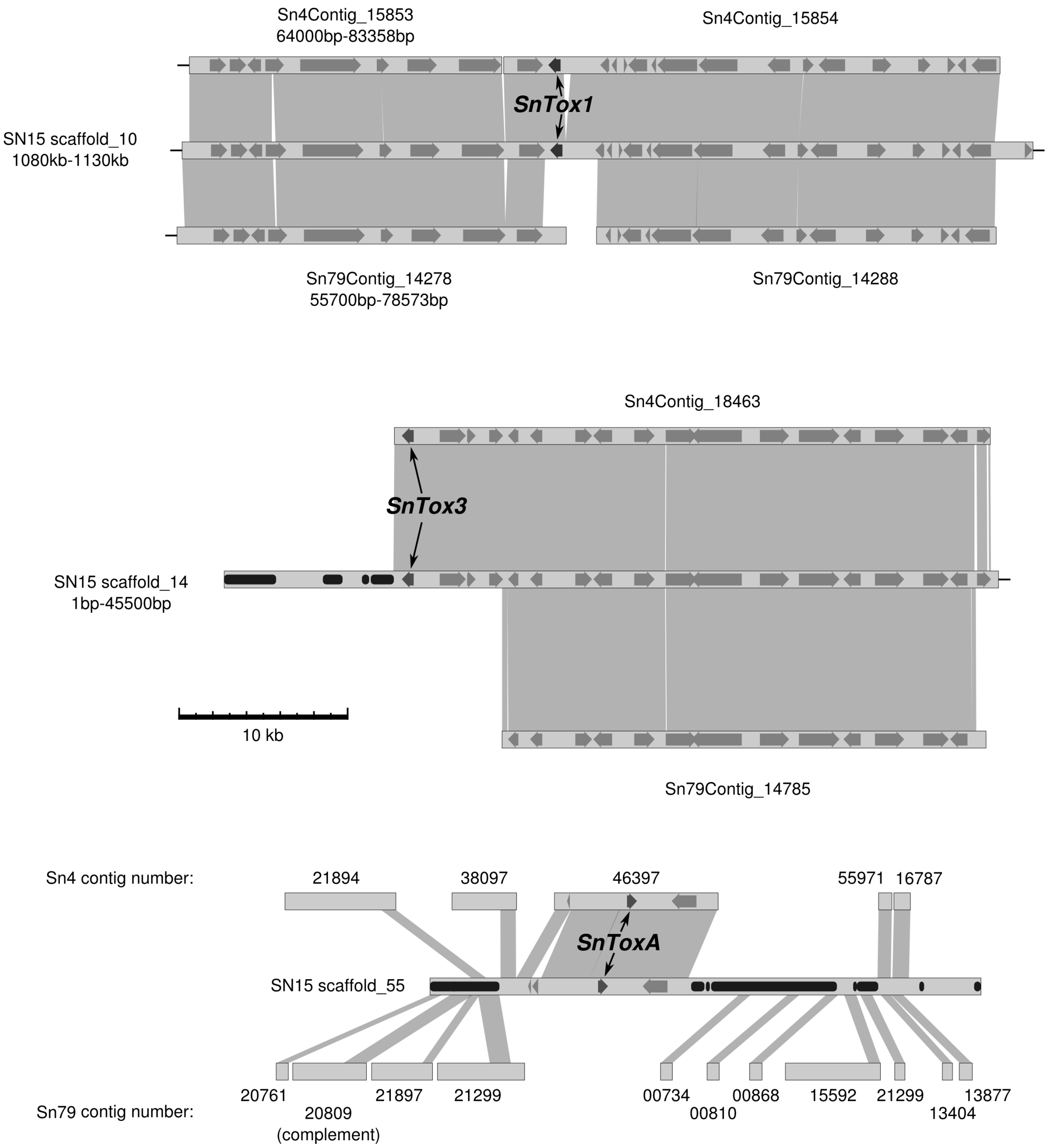

Figure S2 Effector context in three *Stagonospora* strains. Arrows represent genes, lozenges represent repetitive sequence, and boxes represent contigs/scaffolds. Darker trapezoids connecting contigs are blast matches with evalue <= 1e-20. The sequence surrounding *SnTox1* is present in all three strains. *SnTox3* is absent from Sn79is a section that includes four genes. The entire *SnToxA* region is absent in Sn79. Contigs are short around the *SnToxA* region due to the repetitive content in the surrounding sequence.
