# Supplementary material for: Resequencing and Comparative Genomics of Stagonospora nodorum: Sectional Gene Absence and Effector Discovery
Source: G3 (Bethesda). 2013 Jun 1;3(6):959–69. doi: 10.1534/g3.112.004994 (PMC3689807; doi:10.1534/g3.112.004994)
Supplement: Supporting Information [file supp_g3.112.004994_FigureS3.pdf]

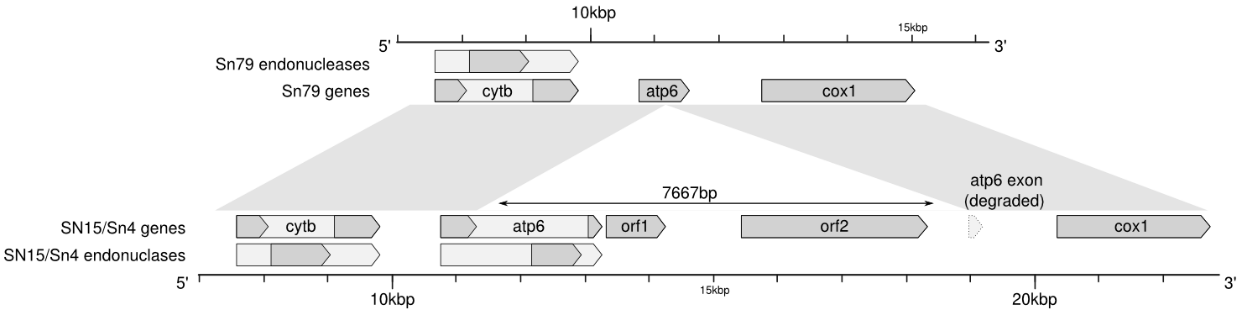

Figure S3 A section of the *S. nodorum* mitochondrial genomes showing that a 7667 bp region present in strains SN15 and Sn4 is absent in Sn79. Regions of homology are shown as darker regions connecting the two sequences. The 7667 bp region includes ORF1 and ORF2, an intronic endonuclease within atp6 and a copy of the 3' *atp6* exon. The insert ends at a degraded copy of the 3' *atp6* exon. This copy differs from the Sn79 *atp6* coding sequence at three SNPs including one nonsense mutation in the degraded Sn15/SN4 copy.
