# Supplementary figures and images for: Resequencing and Comparative Genomics of Stagonospora nodorum: Sectional Gene Absence and Effector Discovery
Source: G3 (Bethesda). 2013 Jun 1;3(6):959–69. doi: 10.1534/g3.112.004994 (PMC3689807; doi:10.1534/g3.112.004994)

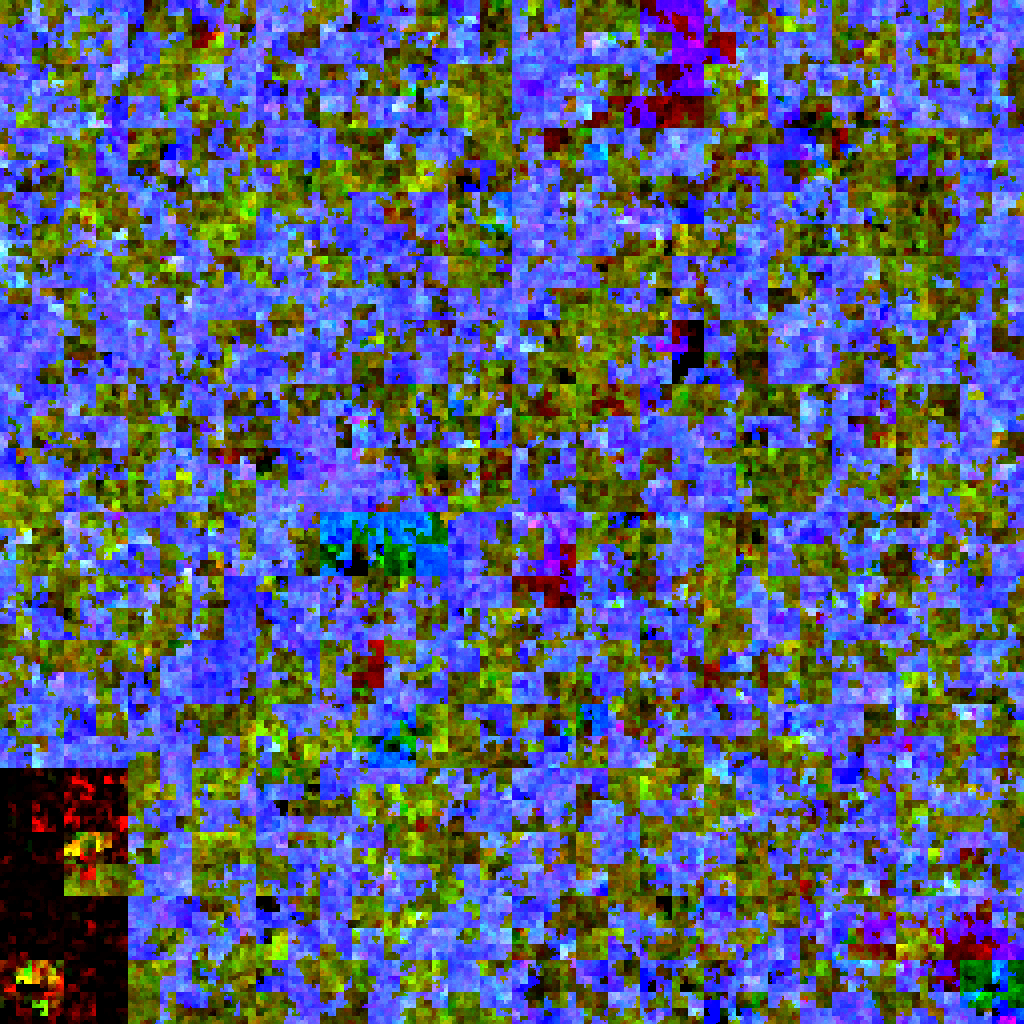

Supplement: Supporting Information [file supp_g3.112.004994_FileS1.zip › FileS1/scaffold_10_10.png]

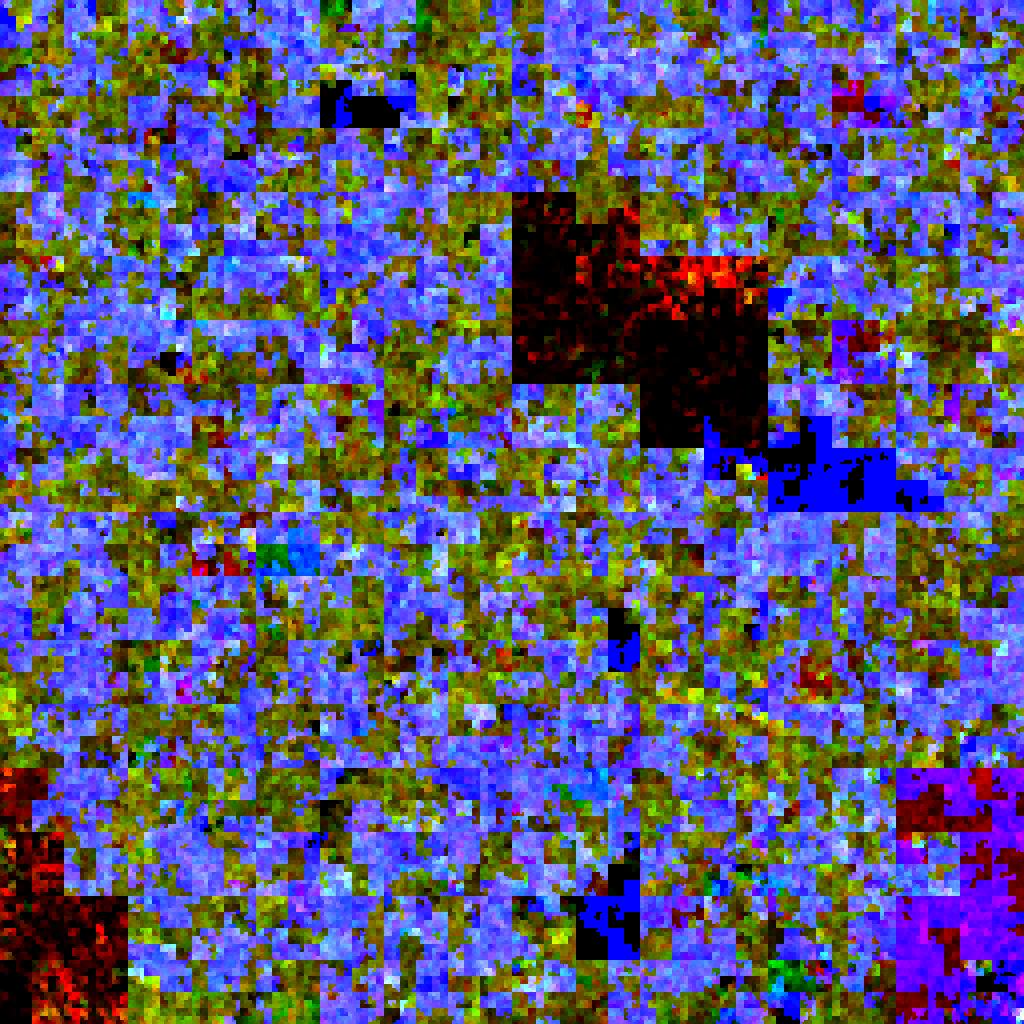

Supplement: Supporting Information [file supp_g3.112.004994_FileS1.zip › FileS1/scaffold_11_10.png]

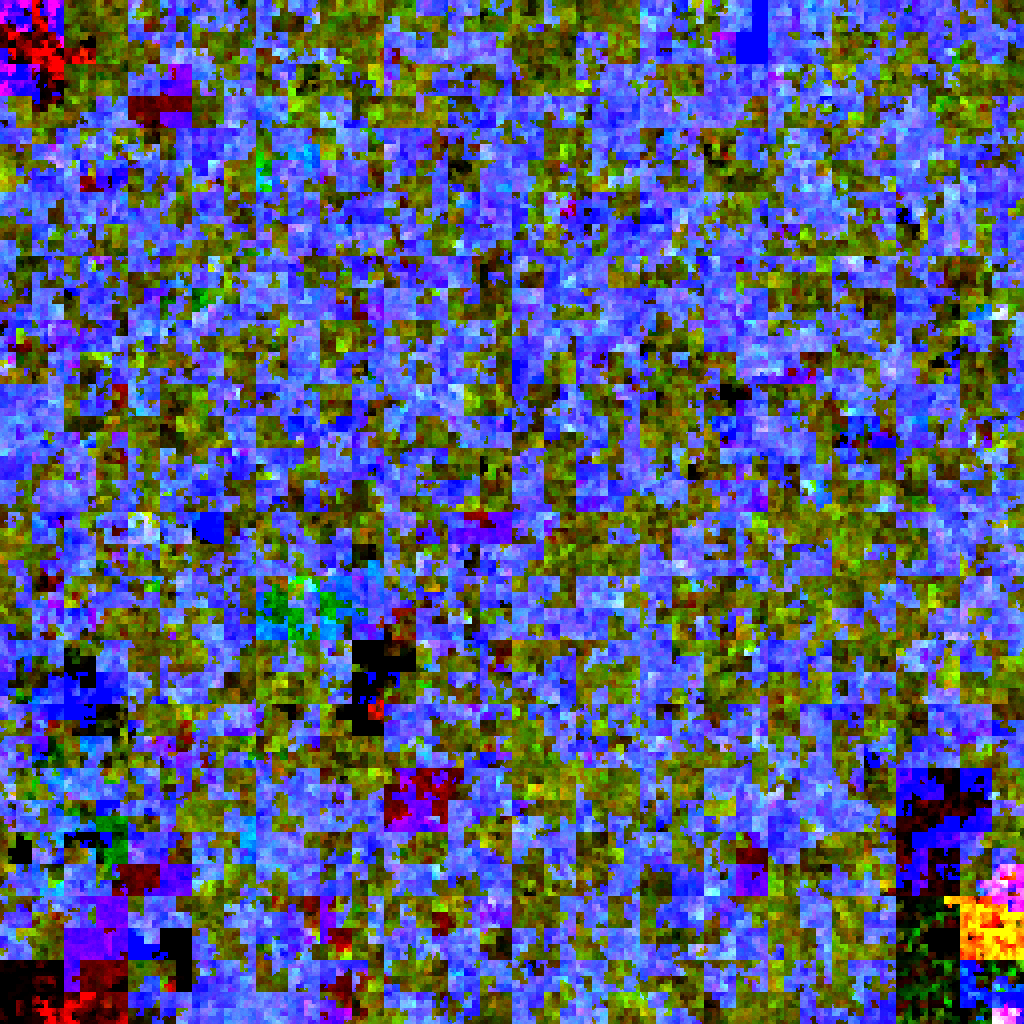

Supplement: Supporting Information [file supp_g3.112.004994_FileS1.zip › FileS1/scaffold_12_10.png]

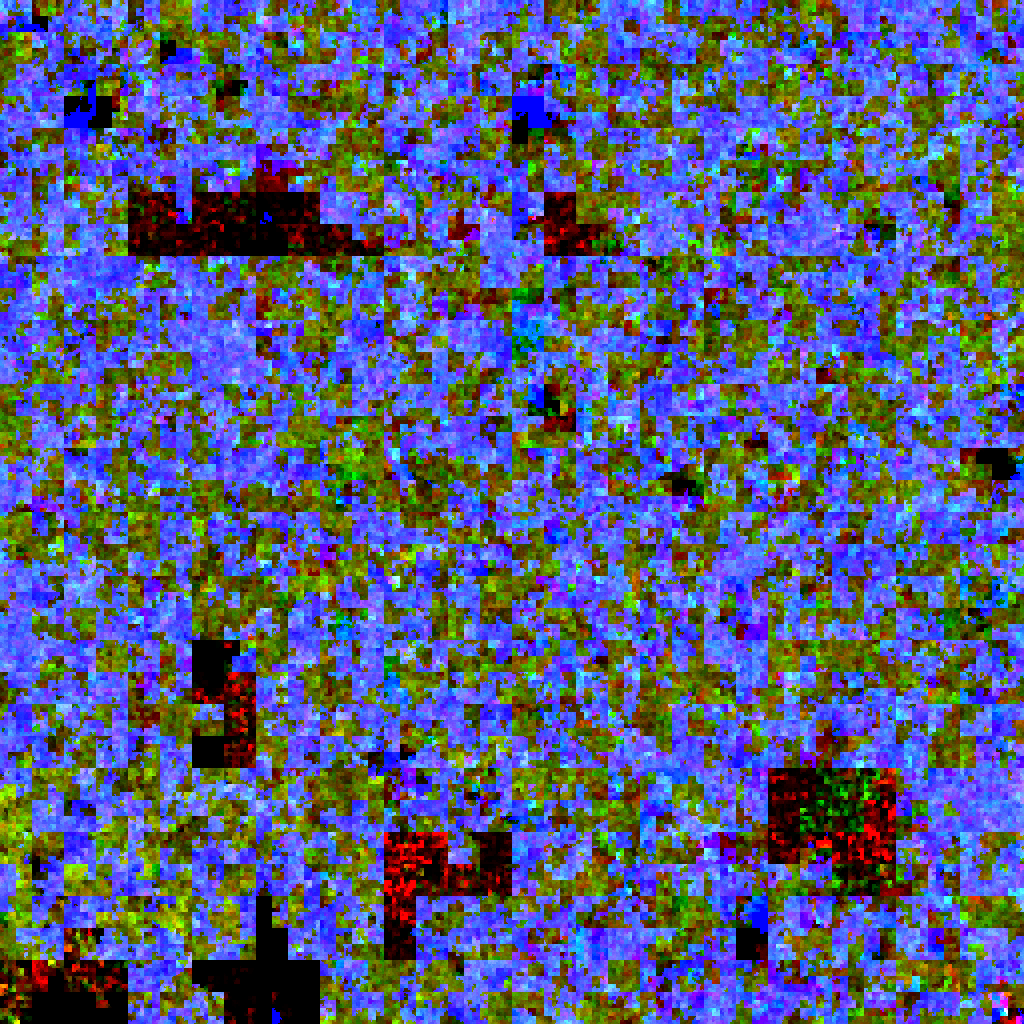

Supplement: Supporting Information [file supp_g3.112.004994_FileS1.zip › FileS1/scaffold_1_10.png]

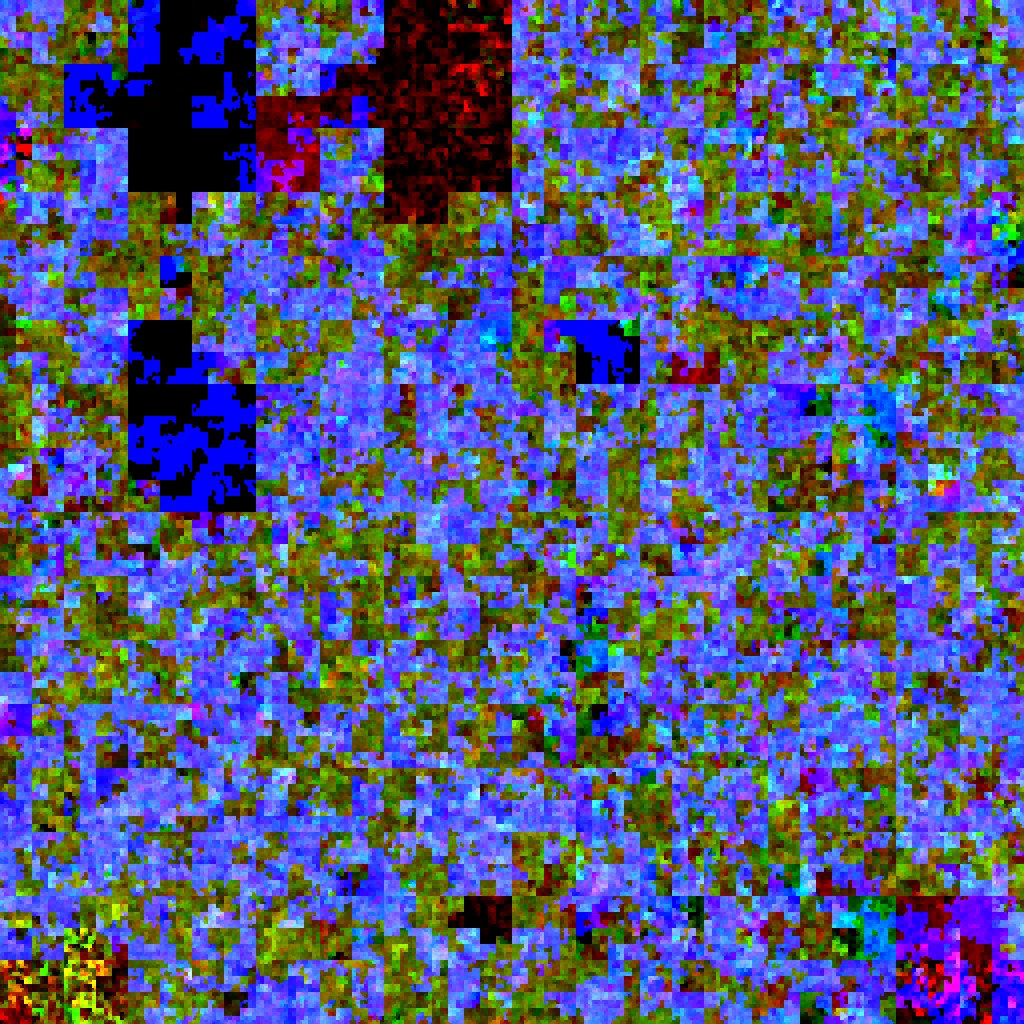

Supplement: Supporting Information [file supp_g3.112.004994_FileS1.zip › FileS1/scaffold_2_10.png]

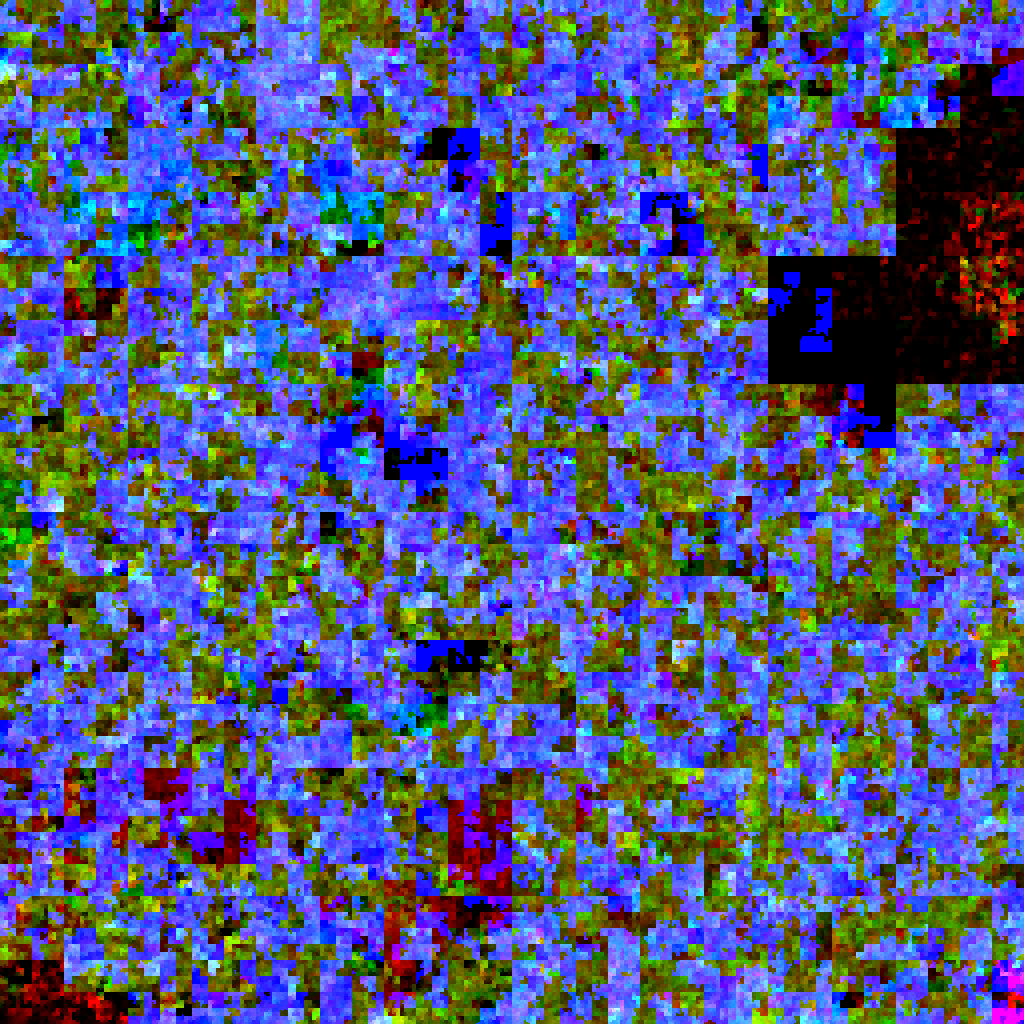

Supplement: Supporting Information [file supp_g3.112.004994_FileS1.zip › FileS1/scaffold_3_10.png]

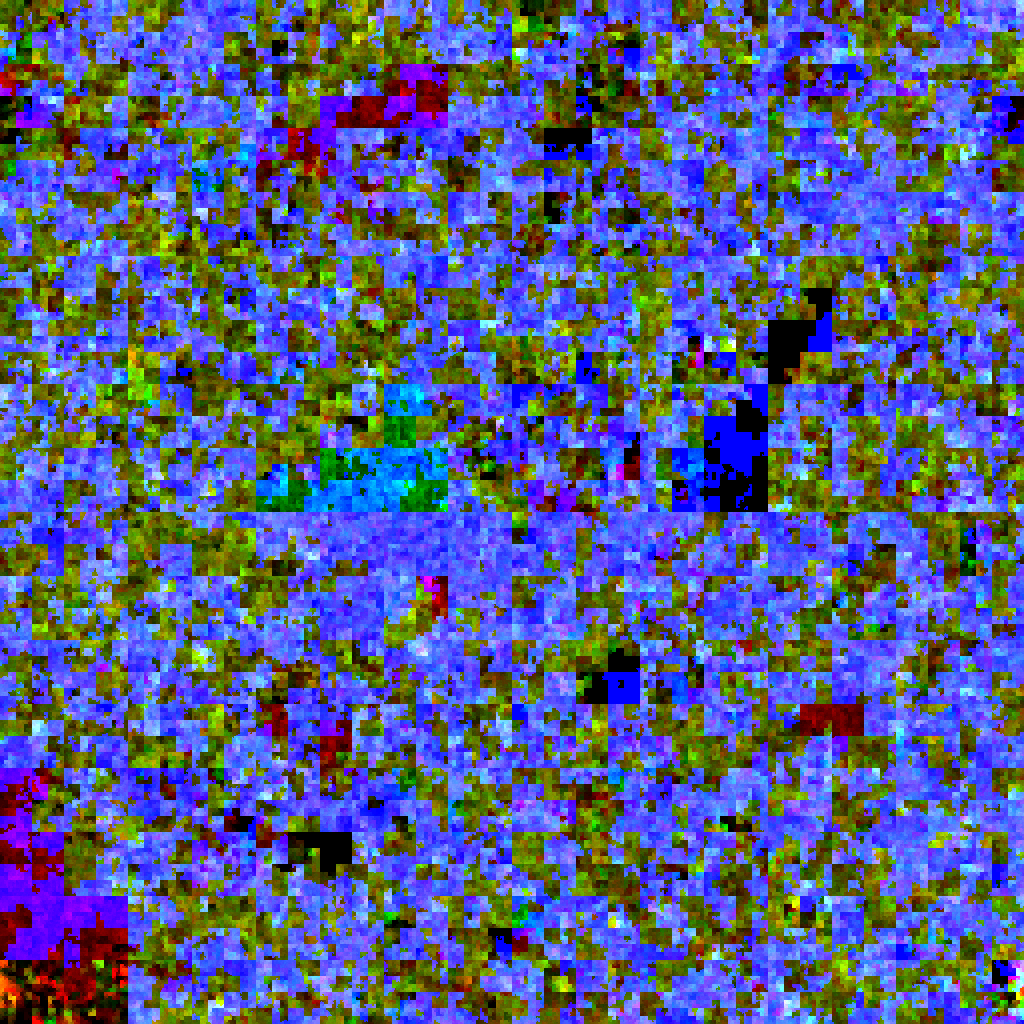

Supplement: Supporting Information [file supp_g3.112.004994_FileS1.zip › FileS1/scaffold_4_10.png]

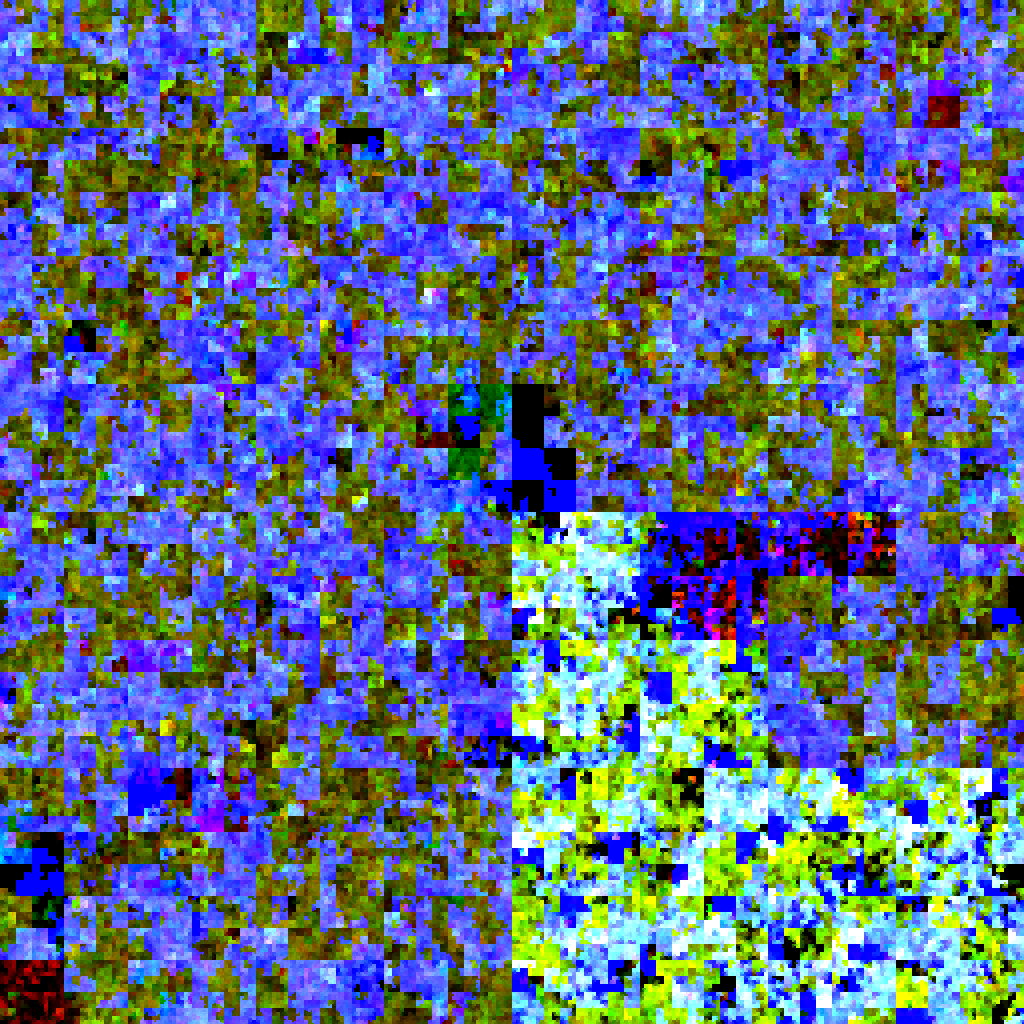

Supplement: Supporting Information [file supp_g3.112.004994_FileS1.zip › FileS1/scaffold_5_10.png]

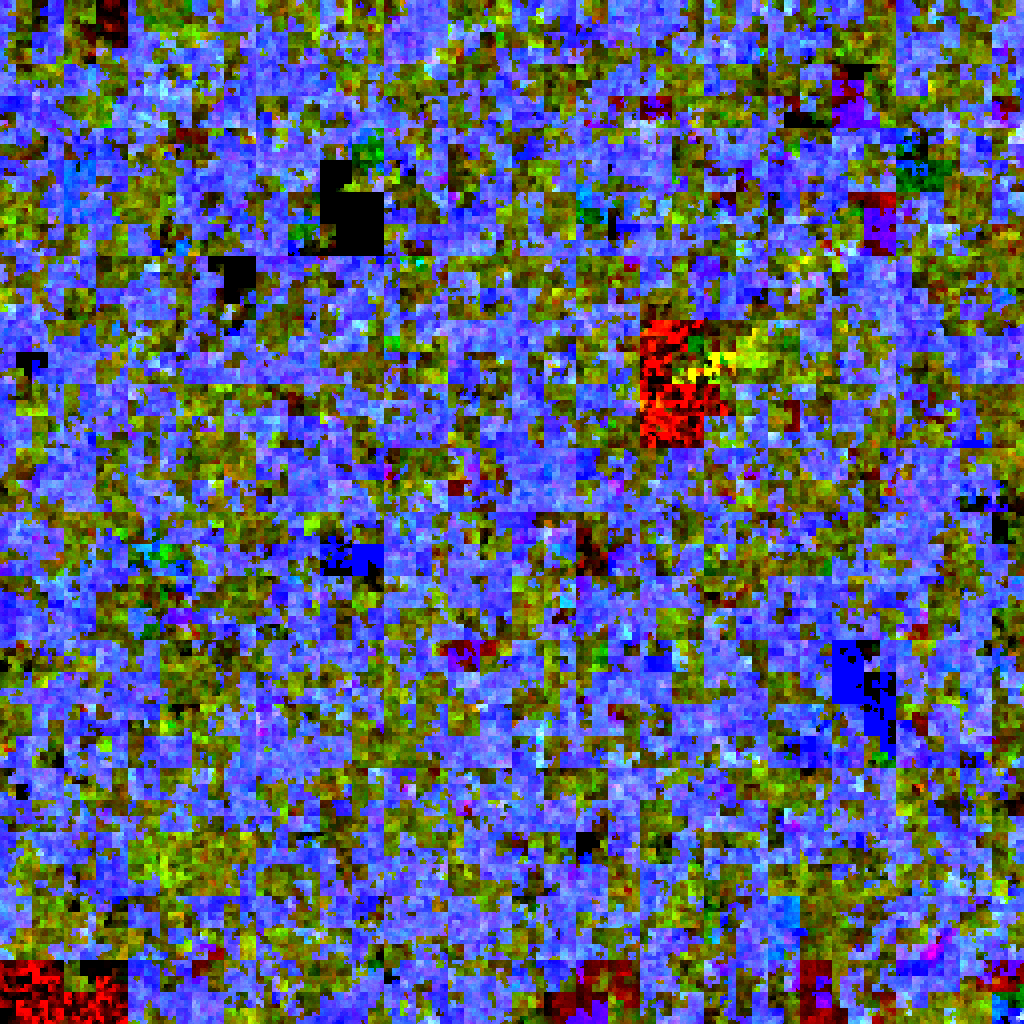

Supplement: Supporting Information [file supp_g3.112.004994_FileS1.zip › FileS1/scaffold_6_10.png]

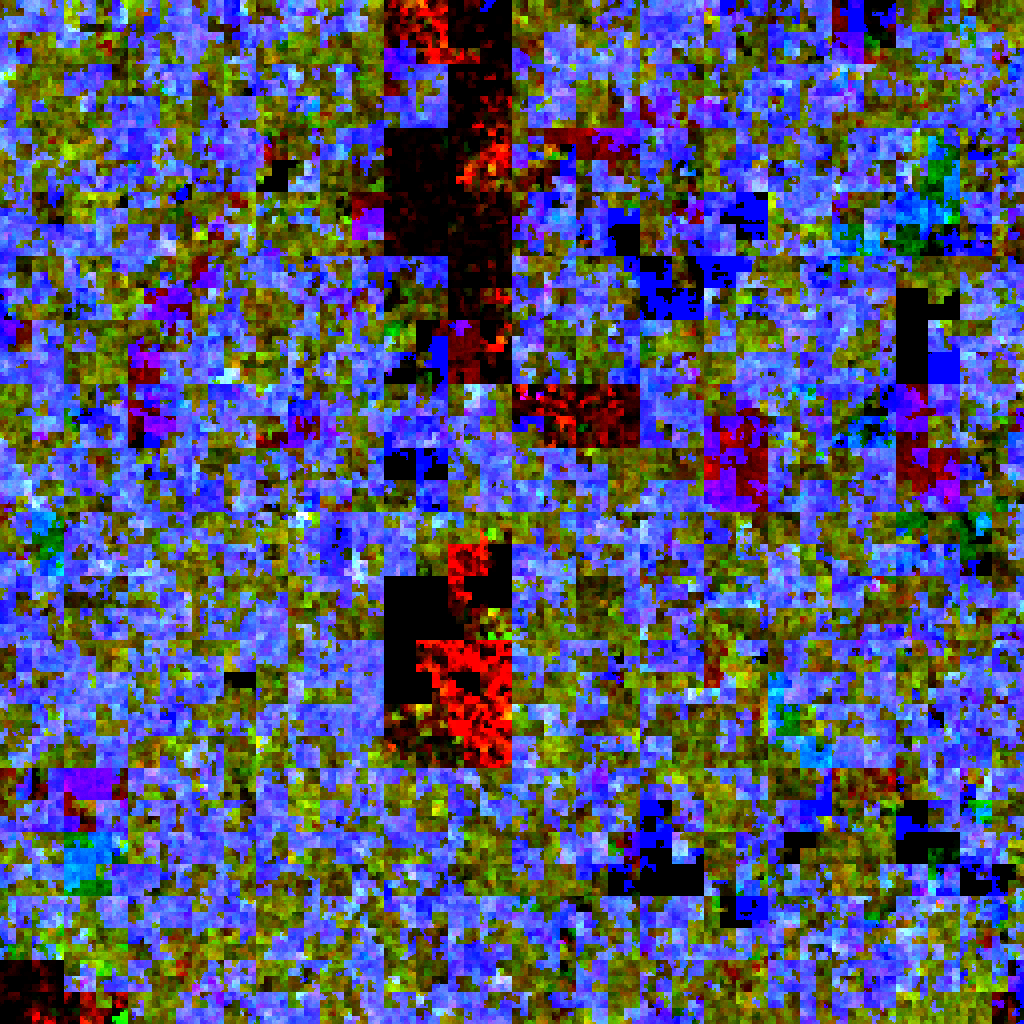

Supplement: Supporting Information [file supp_g3.112.004994_FileS1.zip › FileS1/scaffold_7_10.png]

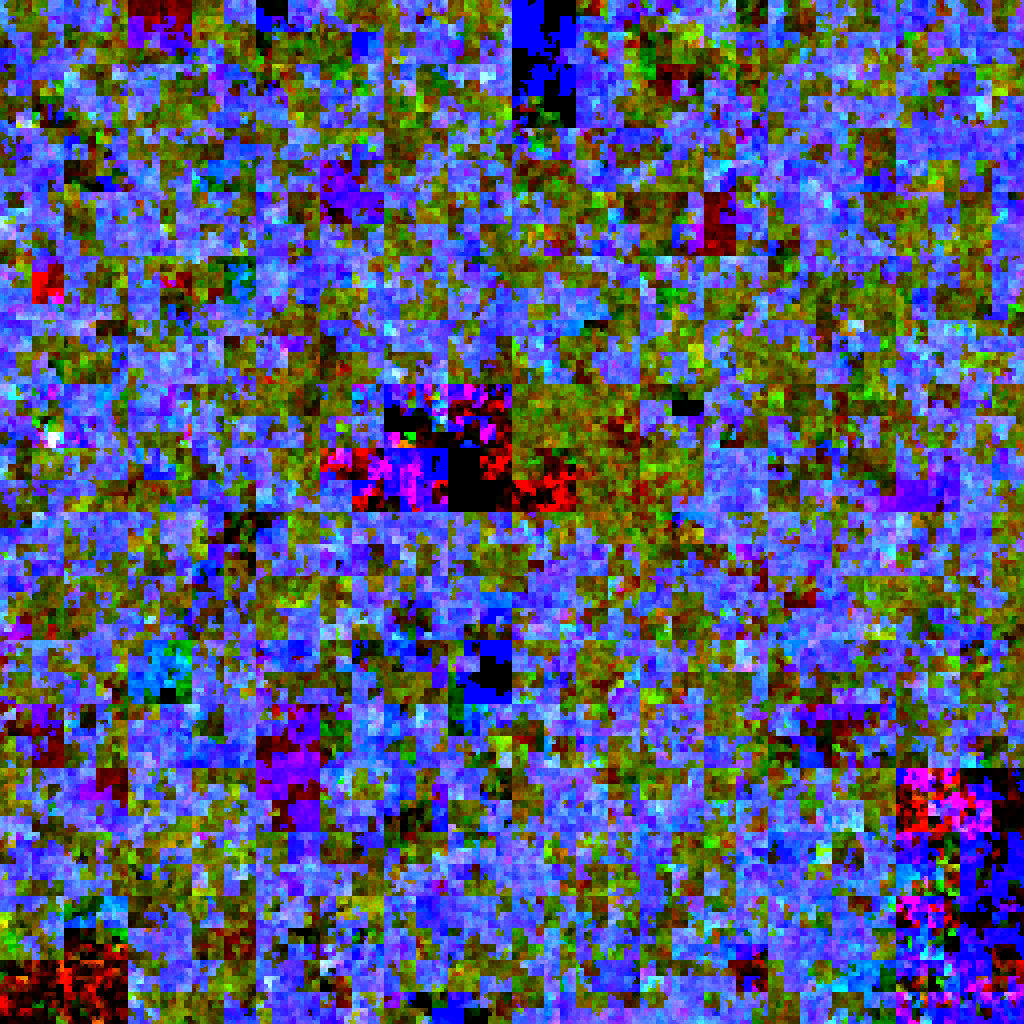

Supplement: Supporting Information [file supp_g3.112.004994_FileS1.zip › FileS1/scaffold_8_10.png]

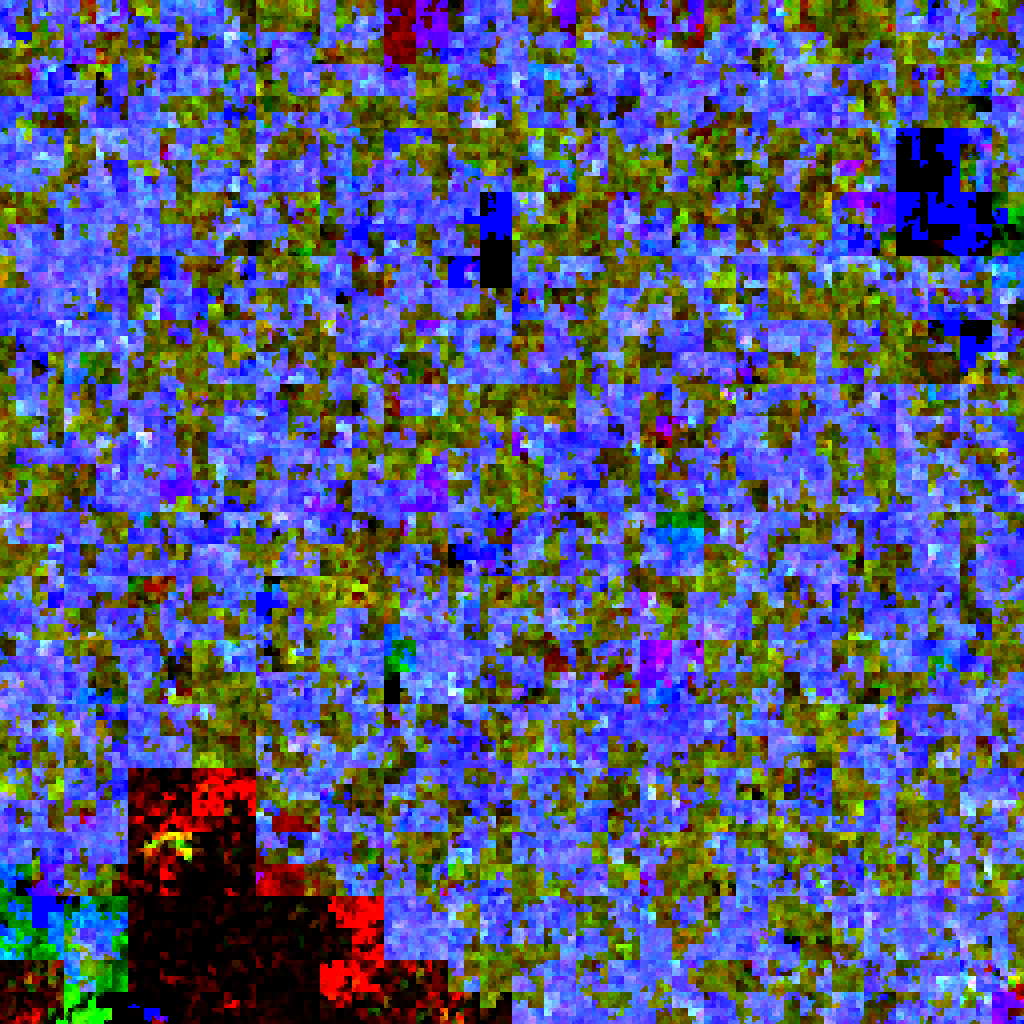

Supplement: Supporting Information [file supp_g3.112.004994_FileS1.zip › FileS1/scaffold_9_10.png]
